# Pretargeted radioimmunotherapy and SPECT imaging of peritoneal carcinomatosis using bioorthogonal click chemistry: probe selection and first proof-of-concept

**DOI:** 10.7150/thno.35461

**Published:** 2019-09-19

**Authors:** Aurélie Rondon, Sébastien Schmitt, Arnaud Briat, Nancy Ty, Lydia Maigne, Mercedes Quintana, Rosemery Membreno, Brian M. Zeglis, Isabelle Navarro-Teulon, Jean-Pierre Pouget, Jean-Michel Chezal, Elisabeth Miot-Noirault, Emmanuel Moreau, Françoise Degoul

**Affiliations:** 1 Université Clermont Auvergne, Imagerie Moléculaire et Stratégies Théranostiques, BP 184, F-63005 Clermont-Ferrand, France. Inserm, U 1240, F-63000 Clermont-Ferrand, France. Centre Jean Perrin, F-63011 Clermont-Ferrand, France.; 2 Institut de Recherche en Cancérologie (IRCM), U1194 - Université Montpellier - ICM, Radiobiology and Targeted Radiotherapy, 34298 Montpellier cedex 5.; 3 Laboratoire de Physique de Clermont, UMR 6533 CNRS/IN2P3, Université Clermont Auvergne, 63178 - Aubière Cedex; 4 Department of Chemistry, Hunter College, City University of New York, New York, NY, USA 10028; 5 Department of Radiology, Memorial Sloan Kettering Cancer Center, New York, NY, USA 10028

**Keywords:** Pretargeting, bioorthogonal chemistry, peritoneal carcinomatosis, therapy, SPECT-CT imaging

## Abstract

***Rationale***: Pretargeted radioimmunotherapy (PRIT) based upon bioorthogonal click chemistry has been investigated for the first time in the context of peritoneal carcinomatosis using a CEA-targeting 35A7 mAb bearing *trans*-cyclooctene (TCO) moieties and several ^177^Lu-labeled tetrazine (Tz) radioligands. Starting from three Tz probes containing PEG linkers of varying lengths between the DOTA and Tz groups (*i.e*. PEG_n_ = 3, 7, or 11, respectively, for Tz-**1**, Tz-**2**, and Tz-**3**), we selected [^177^Lu]Lu-Tz**-2** as the most appropriate for pretargeted SPECT imaging and demonstrated its efficacy in tumor growth control. ***Methods:*** An orthotopic model of peritoneal carcinomatosis (PC) was obtained following the intraperitoneal (i.p.) injection of A431-CEA-Luc cells in nude mice. Tumor growth was assessed using bioluminescence imaging. Anti-CEA 35A7 mAb was grafted with 2-3 TCO *per* immunoglobulin. Pretargeted SPECT imaging and biodistribution experiments were performed to quantify the activity concentrations of [^177^Lu]Lu-Tz-**1-3** in tumors and non-target organs to determine the optimal Tz probe for the PRIT of PC. ***Results:*** The pharmacokinetic profiles of [^177^Lu]Lu-Tz-**1-3** alone were determined using both SPECT imaging and biodistribution experiments. These data revealed that [^177^Lu]Lu-Tz-**1** was cleared *via* both the renal and hepatic systems**,** while [^177^Lu]Lu-Tz-**2** and [^177^Lu]Lu-Tz-**3** were predominantly excreted *via* the renal system. In addition, these results illuminated that the longer the PEG linker, the more rapidly the Tz radioligand was cleared from the peritoneal cavity. The absorbed radiation dose corresponding to pretargeting with 35A7-TCO followed 24 h later by [^177^Lu]Lu-Tz-**1-4** was higher for tumors following the administration of [^177^Lu]Lu-Tz-**2** (*i.e*. 0.59 Gy/MBq) compared to either [^177^Lu]Lu-Tz-**1** (*i.e*. 0.25 Gy/MBq) and [^177^Lu]Lu-Tz-**3** (*i.e*. 0.18 Gy/MBq). In a longitudinal PRIT study, we showed that the i.p. injection of 40 MBq of [^177^Lu]Lu-Tz-**2** 24 hours after the systemic administration of 35A7-TCO significantly slowed tumor growth compared to control mice receiving only saline or 40 MBq of [^177^Lu]Lu-Tz-**2** alone. *Ex vivo* measurement of the peritoneal carcinomatosis index (PCI) confirmed that PRIT significantly reduced tumor growth (PCI = 15.5 ± 2.3 after PRIT *vs* 30.0 ± 2.3 and 30.8 ± 1.4 for the NaCl and [^177^Lu]Lu-Tz-**2** alone groups, respectively). ***Conclusion***: Our results clearly demonstrate the impact of the length of PEG linkers upon the biodistribution profiles of ^177^Lu-labeled Tz radioligands. Furthermore, we demonstrated for the first time the possibility of using bioorthogonal chemistry for both the pretargeted SPECT and PRIT of peritoneal carcinomatosis.

## Introduction

Radioimmunotherapy (RIT) is an effective approach for the treatment of non-solid tumors − such as non-Hodgkin lymphomas 1 − that increases overall survival in patients exhibiting chemoresistance to tyrosine kinase inhibitors. However, the clinical use of RIT remains limited for solid tumors because of the low penetration of radiolabeled monoclonal antibodies (mAbs) and the hematotoxicity caused by their long half-life in the blood 2. To circumvent these drawbacks, pretargeted radioimmunotherapy (PRIT) strategies have been developed that are predicted on the administration of the radiolabeled probe and the mAb in two distinct steps 3.

The oldest PRIT approaches, including systems based on (strept)avidin-biotin 4 and bispecific antibodies (bsAbs) 5, have been evaluated in clinical trials. Unlike the (strept)avidin-biotin system − which demonstrated significant toxicity due to the immunogenicity of (strept)avidin as well as its non-specific interaction with endogenous biotin 6 − the bsAb-based approach has successfully reached phase II/III trials 7. Nonetheless, bsAbs remain difficult to engineer, limiting their widespread clinical implementation. Other systems relying on the use of oligonucleotides (including phosphorodiamidate morpholinos (MORFs) and peptide nucleic acids (PNAs) 8) have attracted interest for *in vivo* imaging 9,10, but significant non-specific uptake in both the kidneys and liver impedes their clinical translation.

The most recently developed approach to PRIT uses bioorthogonal chemistry to facilitate the *in vivo* ligation of mAbs and radiolabeled probes through the formation of a covalent bond between two entities 11. The term 'bioorthogonal' is used to describe chemical reactions that occur under physiological conditions *in vitro* and* in vivo* (pH and temperature) without interfering with biological molecules. Of the various bioorthogonal reactions that have been studied over the past two decades, the inverse-electron demand Diels-Alder cycloaddition (IEDDA) is the most rapid, with high second order rate constants ranging from 10^3^ to 10^6^ M^-1^s^-1^ 12,13. The IEDDA cycloaddition occurs between a dienophile such as a *trans*-cyclooctene (TCO) and a diene like 1,2,4,5-tetrazine (Tz), leading to the formation of a covalent cycloadduct 11. Pretargeting using IEDDA has been successfully applied to both PET/SPECT imaging and PRIT in mice bearing subcutaneous tumors 14-18. However, to the best of our knowledge, no one has reported the use of this approach to pretargeting in disseminated tumors.

Colorectal cancer (CRC) represents the third most common cancer in the world and is associated with a high propensity to generate liver, lung, and peritoneal metastases 19,20. Peritoneal carcinomatosis (PC) is characterized by the progressive invasion of the peritoneal cavity by tumors of various origins, such as ovarian, colorectal, gastric, and, less frequently, non-digestive cancers 21. Metastatic CRC leads to a PC in about 30% of cases, considering both synchronous and metachronous diseases 22 and is often associated with poor prognosis 23. The therapeutic regimen for PC consists of surgery that can be associated with chemotherapy administrated either systemically or intraperitoneally 23. In addition, heated chemotherapy applied in the peritoneal cavity intra-operatively (hyperthermic intraperitoneal chemotherapy; HIPEC) can potentiate the absorption of cytotoxic drugs 24,25.

Despite improvements to the median and overall survival times obtained by combining cytoreductive surgery and HIPEC, both direct morbidity and general mortality remain high, suggesting that the therapy of PC needs to be optimized 26. RIT of metastatic CRC has already demonstrated some effectiveness in preclinical models 27-30. Furthermore, a recent protocol consisting of briefly administering RIT intraperitoneally showed significant efficacy for tumor growth control 31,32. Based on these data, we assume that an innovative approach to PRIT featuring the local intraperitoneal injection of a radiolabeled Tz can be harnessed to increase the efficacy of the treatment of PC.

In the present study, pretargeting was conducted in mice bearing orthotopic tumors (A431-CEA-Luc cells) with a non-internalizing anti-CEA mAb (35A7) 33 in conjunction with several different Tz probes radiolabeled with lutetium-177 (half-life of 6.65 days, Maximal β^-^ energy of 498.3 keV). Three different Tz-based probes (**Table 1,** [^177^Lu]Lu-Tz-**1-3**) were assessed *in vivo via* both SPECT imaging and biodistribution experiments in order to determine the influence of the PEG linker length on their pharmacokinetic and dosimetric profiles. The performance of these three probes was then compared to that of [^177^Lu]Lu-Tz-**4**, which has already demonstrated impressive results in the PRIT of subcutaneous models of pancreatic cancer and CRC 17,18. Based on these biodistribution data, a PRIT study was next conducted with [^177^Lu]Lu-Tz-**2**. Both *in vivo* bioluminescence imaging and the *ex vivo* determination of the peritoneal carcinomatosis index (PCI) revealed a significant slow-down of tumor growth in the treated mice compared to control cohorts. We thus successfully performed the first proof-of-concept pretargeted SPECT imaging and PRIT of PC using the IEDDA cycloaddition.

## Materials and Methods

***Cell line and antibody.***The A431-CEA-Luc is derived from vulvar squamous epithelial carcinoma and was transfected with constructs encoding for both carcinoembryonic antigen (CEA) and luciferase. CEA is highly expressed in human colon carcinoma 34,35. Thus, the abilities of the A431-CEA-Luc cell line to express CEA and disseminate when injected intraperitoneally in nude mice make it a suitable model for mimicking colorectal peritoneal carcinomatosis 33,36. A431-CEA-Luc cells and the non-internalizing murine anti-CEA mAb 35A7 (IgG1) were both provided by Dr. J-P. Pouget and Dr. I. Navarro-Teulon (IRCM, Inserm, Montpellier, France) 33. The cell line was maintained at 37 °C with 5% CO_2_ in humidified environment in Dulbecco's Modified Eagle F12 Medium supplemented with 10% foetal serum, 1% penicillin/streptomycin and extemporaneously with 1% geneticin, 1% hygromycin.

***mAb-TCO conjugation.***The conjugation of TCO-NHS to 35A7 was performed as previously described 37. The quantification of the number of TCO moieties grafted *per* mAb was determined by Matrix Assisted Laser Desorption Ionization Time-of-Flight (MALDI-TOF) mass spectrometry, carried out on a MALDI-TOF/TOF Autoflex Speed (Bruker Daltonics). Sample preparation, sample deposits on the MALDI-TOF target, calibration, and treatment of spectra were performed as previously described 37. Spectrum acquisition was performed in the positive linear ion mode with an ion source voltage of 19.00 kV, a laser power of 75% and smart-beam set at 1 minimum with a frequency of 1000 Hz. A number of 1000 shots was averaged for each spectrum, in the mass-range between 30 and 210 kDa.

***Tz Syntheses.***Tz-**4** was kindly provided by Dr. B. M. Zeglis and Dr. R. Membreno (Hunter College, New York, USA) 18. The syntheses of Tz-**1-3** are detailed in the *Supplementary Information*.

***Radiolabeling.***Tz-**4** was radiolabeled with lutetium-177 as previously described 18. For Tz-**1** and Tz-**2**, two different radiolabeling methods were assessed (*i.e*. Method A and Method B), while Tz-**3** was radiolabeled using only Method B. The first biodistribution experiment with Tz-**1** was performed using radiotracer synthesized using Method A, while the second biodistribution experiment comparing Tz-**1-4** as well as the therapy study with Tz-**2** were performed using radiotracers synthesized using Method B. Both radiolabeling methods are detailed in the *Supplementary Information*.

***Animals.***This investigation conforms to the *Guide for Care and Use of Laboratory Animals published by the US National Institutes of Health* (NIH Publication n°85-236, revised 1996). In addition, all experiments were performed in accordance with the relevant guidelines and regulations and were approved by both the local Ethic committee of Clermont-Ferrand (CEMEAA n°002) and the French Ministry of Education and Research (approval n°5103-2016042010209100). A total of 138 female mice (Nude NMRI Foxn1^nu^/Foxn1^nu^) acquired from Janvier Labs (Le Genest-Saint-Isles, France) were used for the whole experiments. Mice (5 weeks old, median weight 22 g) were housed in standard conditions (n = 5 per cage) in ventilated racks with a 21-24 °C environment with 60% humidity and a 12 h light / 12 h dark cycle with access to food and water *ad libitum*. All i.v. injections were made in the lateral tail vein of vigil mice *via* the dilatation of veins using cotton dipped in hot water to avoid the photochemical isomerization of TCO to *cis*-cyclooctene (CCO). All i.p. injections were made on vigil mice in the lower right quadrant of the abdomen. For *in vivo* imaging, mice were placed under general gas anesthesia receiving isoflurane at 2.5% in oxygen highly enriched air (90%) (Minerve, France). Euthanasia was performed by cervical dislocation after isoflurane gas overdose.

**Biodistributions studies.** Prior to biodistribution experiments, mice were i.p. xenografted with 1×10^6^/ 250 µL A431-CEA-Luc cells. Tumor growth was monitored one day after the graft and 3 days before the graft using bioluminescence imaging *via* the i.p. injection of 15 mg/mL of luciferin (IVIS Spectrum, PerkinElmer, France). Twenty-one days after the graft, the mice were randomly assigned to a protocol group.

*Biodistribution of [^177^Lu]Lu-Tz-**1-3***. 9 mice were randomly divided into three groups (n = 3 *per* group) and were then injected i.p. with 10 MBq of [^177^Lu]Lu-Tz-**1**, [^177^Lu]Lu-Tz-**2**, or [^177^Lu]Lu-Tz-**3**. At 2 h and 24 h after the injection of [^177^Lu]Lu-Tz-**1-3**, the mice were imaged *via* SPECT-CT. The mice were then sacrificed 24 h post injections, and the relevant organs were harvested for gamma counting.

*Selection of the Most Appropriate Tz Radiotracer.* 54 mice were divided into 4 groups (n = 9 or 18 *per* group) and then injected i.p. with 50 µg of 35A7-TCO (≈ 3-4 TCO *per* mAb), followed 24 h later by the injection of [^177^Lu]Lu-Tz-**1-4**. Mice were then sacrificed at 24, 48, and 144 h after the administration of the radioligand, and their main organs were harvested for gamma counting.

*SPECT-CT Imaging.* Multimodal SPECT-CT imaging was performed using a NanoScan SPECT/CT camera (Mediso Ltd) equipped with four detectors and multi pinhole collimation (APT62). Nucline software (Mediso Ltd) was used for image acquisitions and reconstructions (Nucline 3.00.018). CT parameters were as following: helical scan with 480 projections (300 ms *per* projection), 50 kV, 590 uA, pitch 1.0, binning 1:4 and field of view: max. SPECT images were acquired within the CT scan range with a standard resolution. The time *per* projection was determined in accordance with the detected radioactivity (most frequently used: 30 seconds). Mice were placed in a Multicell Mouse L bed (Mediso Ltd) with temperature control (37 °C). SPECT-CT imaging was performed on representative mice (n= 2 or 3) of each group at different time points (*i.e*. 2, 24, 48, and 72 h) after the injection of [^177^Lu]Lu-Tz-**1-3**. SPECT image reconstruction was conducted using TeraTomo3D (Nucline v3.00.018) with normal dynamic range. Regularization filters, reconstruction resolution, and iterations were set to “medium”. Additional corrections were performed during reconstruction: Monte Carlo correction quality was set to “high”; Attenuation: based on CT attenuation map and scatter corrections; Activity decay correction: during acquisition time lapse.

*Dosimetry.* Absorbed doses to organs were calculated for [^177^Lu]Lu-Tz-**1-4** following the MIRD methodology 38. Self S values, calculated using GATE Monte Carlo simulations 39-41, were extracted from a previous study 42 on same mouse models for heart, lungs, liver, kidneys, and spleen. In addition, a specific self S value calculation was performed for a spherical tumor with a mean weight of 0.015 g and a corresponding diameter of 3 mm. As the mean energy of electrons emitted from the beta decay of [^177^Lu] is 133.3 keV, their mean range (0.2 mm) in soft tissues is very small compared to the mouse organ sizes, therefore only self S values have been considered in this study.

**Pretargeted radioimmunotherapy.** Throughout the duration of the experiment, mice were housed individually in armored enclosures with 12 h light / 12 h dark cycles and were provided access to food and water *ad libitum*. Nine days after inoculation, the mice were blindly assigned to 3 groups (n = 6 *per* group). One group was first injected i.v. with 50 µg / 150 µL of 35A7-TCO (≈ 3-4 TCO *per* mAb) followed 24 h later by the i.p. administration of 40 MBq / 250 µL of [^177^Lu]Lu-Tz-**2**. The two other groups were first injected i.v. with 150 µL of 0.9% NaCl, followed 24 h later by the i.p. administration of either 250 µL of 0.9% NaCl or 40 MBq/ 250 µL of [^177^Lu]Lu-Tz-**2**. The longitudinal PRIT study was conducted in a double-blind manner to avoid any bias.

*Tumor growth assessment*. Tumor growth was monitored using bioluminescence imaging one day after xenografting, one day before the first injections, and once a week until the sacrifice of each mouse. Mice were sacrificed in the event of weight loss (≥ 20%), behavioral changes, and the appearance of clinical signs such as anemia, pain, or palpable tumor mass on the abdomen. All observations were reported in a score grid (*i.e*. absence, moderate, severe annotations), and the mice were sacrificed when the maximal ethical disease activity index was reached. Monitoring could not be performed after 20 days of tumor growth due to the lack of linearity between the bioluminescence signal and tumor size 33.

*PCI determination*. We performed the PCI − a standard method allowing quantifying the number of peritoneal tumors through the attribution of a score ranging from 1 to 39, which correlates the severity of the disease − during mice necropsy. PCI was determined according to the score method described by Sugarbaker 24 and adapted for rodents by Klaver *et al.* 43.

**Statistical analysis.** Statistical analyses were performed using XLSTAT 2012 software. Continuous data were expressed as mean ± standard deviation (SEM) and were compared using a one-way, two-way ANOVA followed by Tuckey test. Survival was compared using Kaplan-Meier test. We considered p < 0.05 as statistically significant.

## Results

### Radiolabeling of Tz-1-4 with Lutetium-177

We first radiolabeled Tz-**1** using Method A, which requires 20 min heating at 50 °C, affording the expected [^177^Lu]Lu-Tz-**1** with high radiochemical purity (98%) and molar activity (> 8 GBq/µmol). However, this method provided unreproducible radiochemical yields (≈ 60-80%, n = 15) after purification and formulation due to the formation of an unidentified radiolabeled byproduct (**Table 1**). The radiolabeling of Tz-**2** according to Method A likewise resulted in both low radiochemical yields and purities (36% and 24%, respectively), and the method was unsuccessful for the radiolabeling of Tz-**3** (data not shown). Furthermore, the entire radiolabeling process of Tz-**1** and Tz-**2** − including HPLC purification and formulation − required at least 105 min. Subsequently, the room temperature radiolabeling method described by Membreno *et al*. 18 (*i.e*. Method B; which did not involve a purification step) was tested and led to improvements in the radiochemical yields (79-89% overall yields) and purities (equal or superior to 97%) of [^177^Lu]Lu-Tz-**1-4** after formulation in only 75 min (**Table 1**).

### SPECT imaging of [^177^Lu]Lu-Tz-1-3 alone

**Figure 1:**
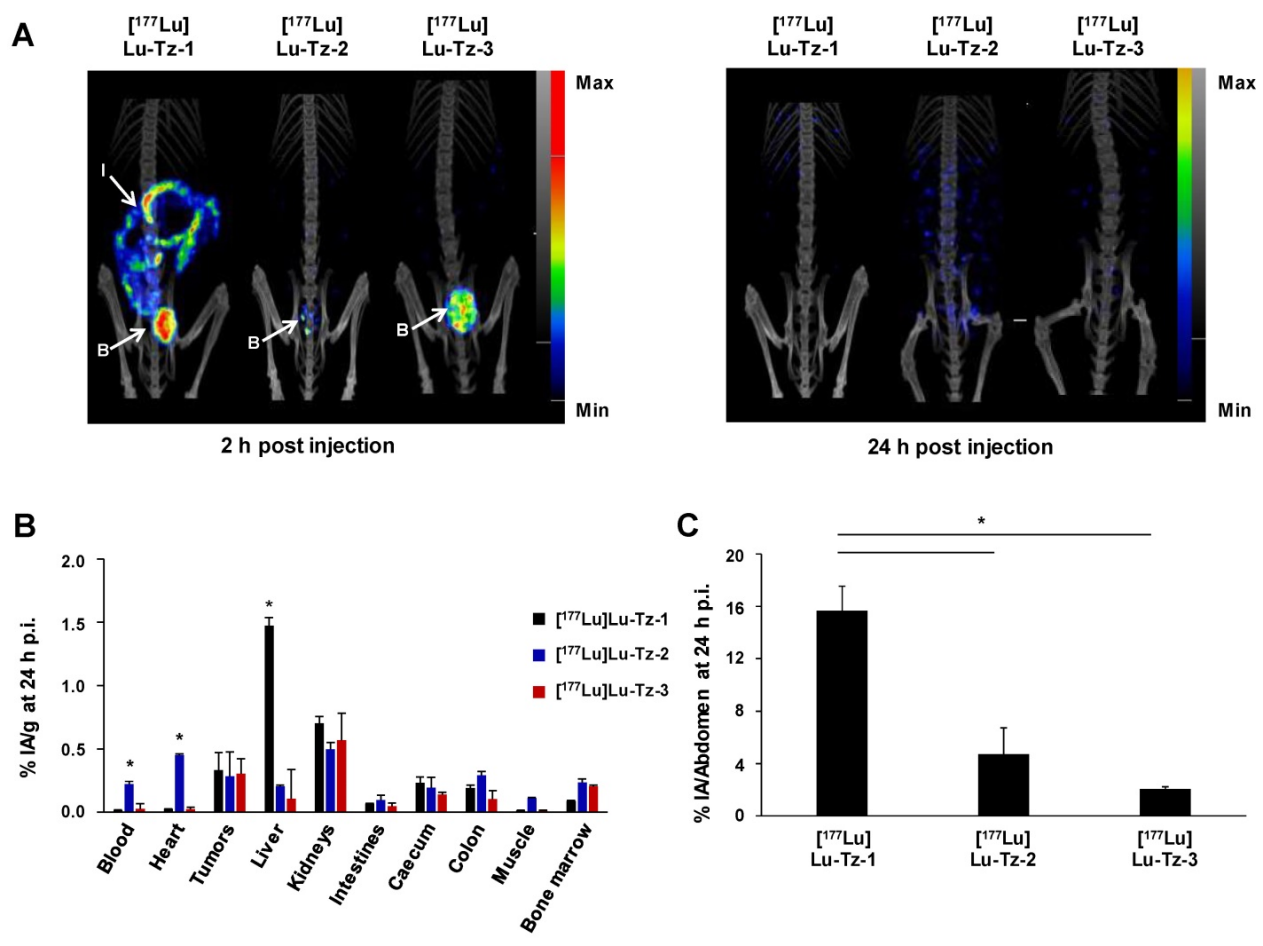
Comparison of the pharmacokinetic profiles of [^177^Lu]Lu-Tz-**1-3** alone in mice bearing A431-CEA-Luc peritoneal disseminated tumors (i.p. injection). (A) SPECT-CT imaging of [^177^Lu]Lu-Tz-**1-3** at 2 h and 24 h p.i. (10 MBq) 21 days post xenograft. (B) %IA/g in organs at 24 h p.i. for [^177^Lu]Lu-Tz-**1-3** (10 MBq). B: bladder, I: intestines. * p < 0.05: [^177^Lu]Lu-Tz-**1** *vs* [^177^Lu]Lu-Tz-**2** and [^177^Lu]Lu-Tz-**3**. (C) %IA in the entire abdomen at 2 h post injection of [^177^Lu]Lu-Tz-**1-3** (10 MBq) using SPECT imaging. * p < 0.05: [^177^Lu]Lu-Tz-**1** *vs* [^177^Lu]Lu-Tz-**2** and [^177^Lu]Lu-Tz-**3** (One-way ANOVA test). Scale bars: SPECT: Max: 4000 kBq/mL, Min: 700 kBq/mL (2 h) and Max: 2000 kBq/mL, Min: 100 kBq/mL (24 h); CT: Max: 4000 HU, Min: 400 HU (at both 2 and 24 h).

The biodistribution profiles of [^177^Lu]Lu-Tz-**1-3** were monitored using SPECT imaging at 2 h and 24 h after i.p. administration. These data clearly demonstrated that without the prior injection of 35A7-TCO, [^177^Lu]Lu-Tz-**2** and [^177^Lu]Lu-Tz**-3** have different pharmacokinetic profiles than [^177^Lu]Lu-Tz-**1** (**Figure 1A**). Indeed, both SPECT imaging and radioactivity counting showed exclusively renal clearance for [^177^Lu]Lu-Tz-**2** and [^177^Lu]Lu-Tz**-3**, while [^177^Lu]Lu-Tz-**1** is primarily eliminated *via* the liver. In addition, [^177^Lu]Lu-Tz-**2** displayed a significantly longer circulation time in the blood compared to the two other radioligands (at 24 h p.i.: 0.22 ± 0.02 %IA/g for [^177^Lu]Lu-Tz-**2*** vs* 0.02 ± 0.00 %IA/g and 0.03 ± 0.04 %IA/g for [^177^Lu]Lu-Tz-**1** and [^177^Lu]Lu-Tz-**3**, respectively) (**Figure 1B**). In contrast, the longer the PEG linker, the more rapidly the Tz-based radioligand was eliminated from the peritoneal cavity. Indeed, 24 hours after the injection of [^177^Lu]Lu-Tz-**1-3**, the %IA in the entire abdomen was significantly higher for [^177^Lu]Lu-Tz-**1** compared to [^177^Lu]Lu-Tz-**2** and [^177^Lu]Lu-Tz-**3** (**Figure 1C**).

### Biodistribution Profiles of [^177^Lu]Lu-Tz-1-4 with Prior Injection of 35A7-TCO

Pretargeted SPECT imaging − performed *via* the i.v. injection of 35A7-TCO, followed 24 h later by the i.p. administration of [^177^Lu]Lu-Tz-**1** − produced a specific signal in peritoneal tumors (**Supplementary Figure S1**). It should be noted that a study using [^177^Lu]Lu-Tz-**1** to compare the effect of the injection route of 35A7-TCO (*i.e*. i.v. or i.p.) on the *in vivo* performance of the system was performed and resulted in no statistical differences between the two different modes of injection (**Supplementary Figure S1B**). Indeed, 48 h after the i.p. administration of [^177^Lu]Lu-Tz-**1**, the tumoral %IA/g reached maxima of 4.07 ± 0.03% and 5.50 ± 0.02%, respectively, with the i.v. and i.p. administration of 35A7-TCO.

**Figure 2:**
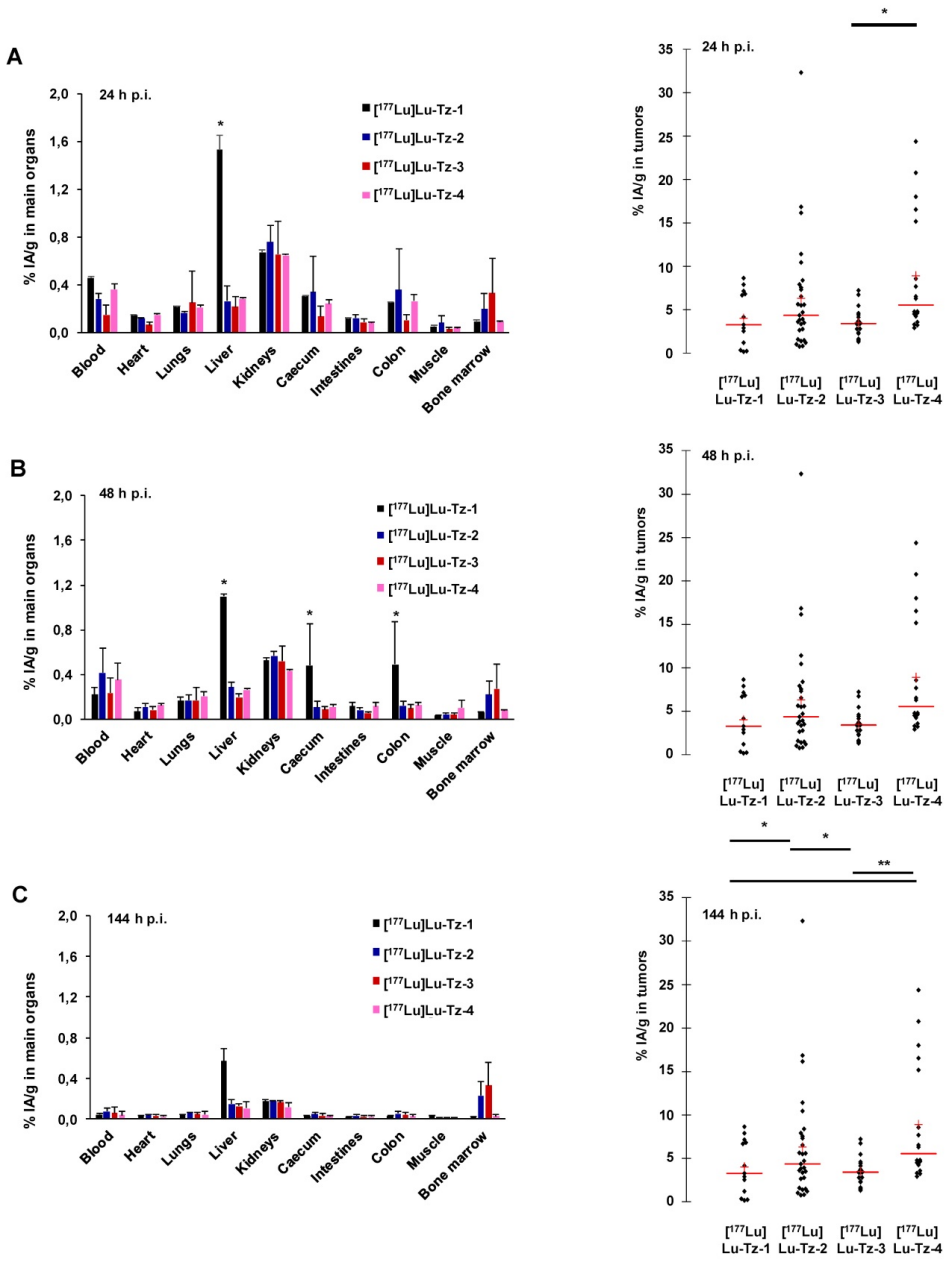
(A-C) Systematic selection of the most appropriate Tz-bearing radioligand for the PRIT of peritoneal carcinomatosis based on *in vivo* performance in mice bearing peritoneal A431-CEA-Luc disseminated tumors. Peritoneal tumors were first injected i.v. with 50 µg of 35A7-TCO, followed 24 h later by the i.p. injection of [^177^Lu]Lu-Tz-**1-4** (10 MBq). %IA/g measured in main organs (left) and in peritoneal tumors (right) harvested at 24 (A), 48 (B) and 144 h (C) post injection. * p < 0.05: [^177^Lu]Lu-Tz-**4** *vs* [^177^Lu]Lu-Tz-**3**, [^177^Lu]Lu-Tz-**2** *vs* [^177^Lu]Lu-Tz-**1** and [^177^Lu]Lu-Tz-**2** *vs* [^177^Lu]Lu-Tz-**3**. ** p < 0.005: [^177^Lu]Lu-Tz-**4** *vs* [^177^Lu]Lu-Tz-**1** and [^177^Lu]Lu-Tz-**4** *vs* [^177^Lu]Lu-Tz-**3** (Tuckey Test).

Pretargeted biodistribution studies of [^177^Lu]Lu-Tz-**1-4** following the injection of 35A7-TCO demonstrated variations in the tumoral activity concentrations produce by the radioligands, with the maximum values for [^177^Lu]Lu-Tz-**2** and [^177^Lu]Lu-Tz-**4** occurring at 24 h p.i (6.29 ± 4.27 %IA/g and 8.88 ± 5.61 %IA/g, respectively) (**Figure 2A-C**). The tumoral %IA/g using [^177^Lu]Lu-Tz-**2** at both 24 and 144 h p.i. was significantly higher than that obtained with either [^177^Lu]Lu-Tz-**1** and [^177^Lu]Lu-Tz-**3** (*i.e*. 6.29 ± 4.27 % for [^177^Lu]Lu-Tz-**2** *vs* 4.00 ± 2.67 % and 3.48 ± 1.20 % for [^177^Lu]Lu-Tz-**1** and [^177^Lu]Lu-Tz-**3**, respectively, at 24 h p.i.; 3.80 ± 2.18 % for [^177^Lu]Lu-Tz-**2** *vs* 1.50 ± 0.60 % and 1.52 ± 0.93 % for [^177^Lu]Lu-Tz-**1** and [^177^Lu]Lu-Tz-**3**, respectively, at 144 h p.i.). However, at each of the three different times p.i., no significant difference in the tumoral activity concentration was observed between [^177^Lu]Lu-Tz-**4** and [^177^Lu]Lu-Tz-**2**.

### Dosimetry Corresponding to Pretargeting Experiments

Dosimetry was calculated for self-organ irradiation taking into account S-factors computed for main organs in the same nude mice species 42.The value of the S-factor for small tumors computed by the same algorithm was found to be 1.32 Gy/Bq/s (**Table 2**). With respect to pretargeting with [^177^Lu]Lu-Tz**-1-4** and 35A7-TCO, the dosimetry values obtained for main organs were low, as expected based on the biodistribution studies. In PRIT experiments, the highest doses to the tumors were provided by [^177^Lu]Lu-Tz-**2** and [*^177^*Lu]Lu-Tz-**4**, with 23.692 ± 20.87 and 25.23 ± 22.38 Gy/40 MBq, respectively.

### Efficiency in PRIT of Peritoneal Carcinomatosis Using [^177^Lu]Lu-Tz-2 and 35A7-TCO

**Figure 3:**
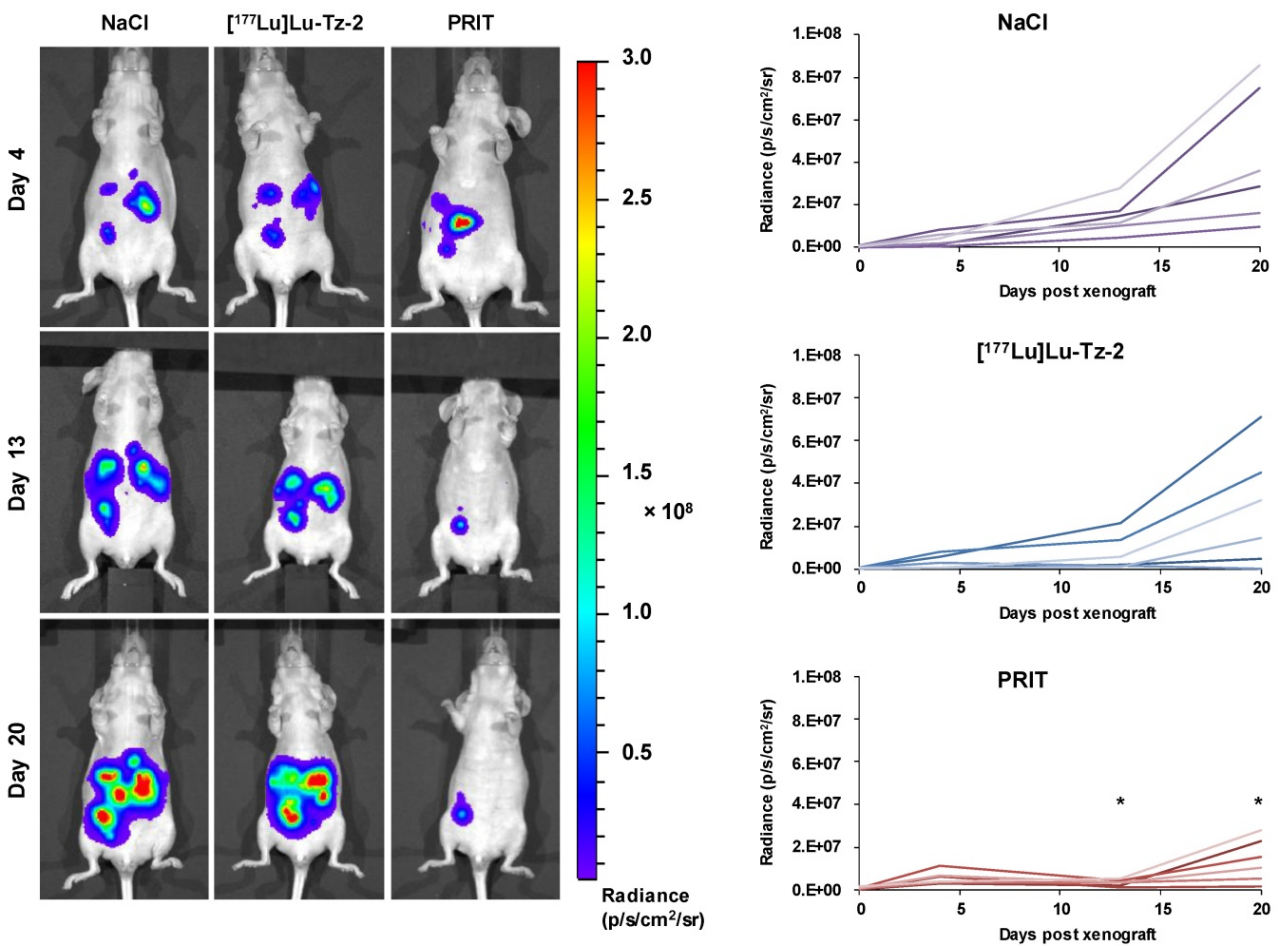
Longitudinal study of the PRIT of PC using [^177^Lu]Lu-Tz-**2** and 35A7-TCO. After 9 days of tumor growth, mice bearing disseminated A431-CEA-Luc tumors were injected with either the treatment or the placebo. Control groups first received an i.v. injection of NaCl followed 24 h later by an i.p. injection of either NaCl or 40 MBq of [^177^Lu]Lu-Tz-**2**. The PRIT group first received an i.v. injection of 50 µg of 35A7-TCO, followed 24 h later by an i.p. injection of 40 MBq of [^177^Lu]Lu-Tz-**2**. Left: *In vivo* bioluminescence imaging at different time points post-treatment. Right: *In vivo* tumor growth was monitored and quantified from 4 to 20 days post-treatment using bioluminescence imaging. *p < 0.05: day 13 and day 20 of PRIT group *vs* day 13 and 20 of NaCl and [^177^Lu]Lu-Tz-**2** control groups (one-way ANOVA test).

**Figure 4:**
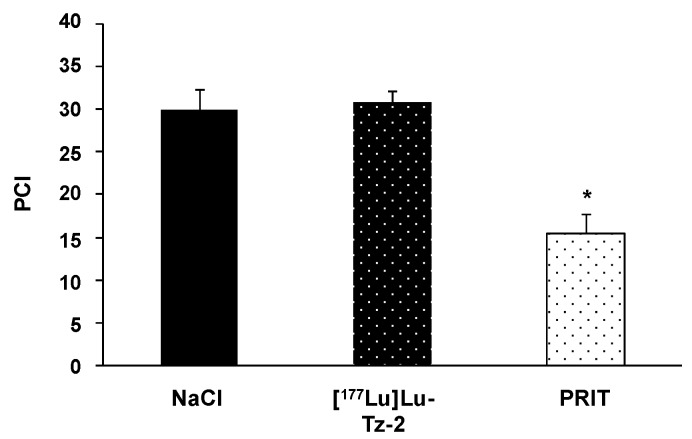
*Ex vivo* determination of the peritoneal carcinomatosis index (PCI) according to the score grid developed by Sugarbaker *et al*. (*24*) and adapted for rodents by *Klaver et al*. (*43*). PCI was measured when the maximal ethical disease activity index of each mouse was reached. * p < 0.05: PRIT *vs* both control groups (One-way ANOVA test). *NB*: mice were assigned randomly to the different groups, and both tumor growth monitoring and mouse health observations were performed in a double-blind manner.

Before beginning the PRIT experiment, we randomized the mice in order to have similar groups in terms of tumor burden (**Supplementary Figure S2**). The bioluminescence imaging follow-up − performed after the injection of either 40 MBq [^177^Lu]Lu-Tz-**2** (with or without prior i.v. injection of 50 µg of 35A7-TCO 24 hours before) or physiological saline NaCl − demonstrated a significant slow-down of tumor progression in the mice from the PRIT cohort compared to the control cohorts after 13 days (**Figure 3**). From 13 to 20 days p.i., the tumors of the PRIT groups started to grow again but did so significantly more slowly than those of the control groups. In addition, the PCI was significantly lower in the mice from the PRIT group compared to the control groups (*i.e*. 15.5 ± 2.3 for PRIT group *vs* 30.0 ± 2.3 and 30.8 ± 1.4 for NaCl and control [^177^Lu]Lu-Tz-**2**, respectively), a finding which correlates with the *in vivo* observations (**Figure 4**).

## Discussion

Pretargeted radioimmunotherapy based on biorthogonal click chemistry has been used for the first time in the context of peritoneal carcinomatosis using a CEA-targeted 35A7 mAb grafted with TCO and several different Tz constructs radiolabeled with lutetium-177. Two protocols for radiolabeling were used with Tz-**1-3**. Method A, described by Robillard and co-workers, involves a heating step 14. Despite leading to high molar activities and excellent radiochemical purities, this 3-step process − radiolabeling, HPLC purification, and formulation − provided moderate and unreproducible radiochemical yields due to the formation of an unidentified radiolabeled byproduct that necessitated a HPLC purification step. The formation of this byproduct extended the duration of the synthesis and formulation process and led to a loss of the desired radiolabeled compound. In contrast, Method B, described by Membreno *et al*. 18, facilitated the quick and efficient radiolabeling of Tz-**1-4** at room temperature with high molar activities, high radiochemical yields, and purities. Critically, we did not observe the formation of any radiolabeled byproducts using Method B.

PC is commonly managed by the surgical cytoreduction of macroscopic lesions 23 which can be accompanied with intraperitoneal chemotherapy 19. The effectiveness of surgery mainly relies on the complete removal of tumor tissue, which in turn is directly correlated with the ability to visualize microscopic lesions. In a previous study, we demonstrated the possibility of using fluorescent Tz probes to detect PC tumors 37. Recent developments in efficient fluorescent dyes raises the possibility of using a TCO/Tz-based system in a *per*-operatory setting as a guide to help surgeons detect microscopic lesions 44. On the other hand, brief intraperitoneal RIT − which consists of the i.p. injection of radioimmunoconjugates with high molar activity for 30-60 min, followed by the extensive washing of the peritoneal cavity with a saline solution using a peristaltic pump − has recently proven effective in PC of either colorectal 31 or ovarian 32 origins. Pretargeting using the inverse electron demand Diels-Alder ligation could thus provide a promising theranostic strategy for this disease. In the present study, we therefore investigated the efficacy of both the pretargeted SPECT and PRIT of PC using bioorthogonal click chemistry.

Publications on *in vivo* pretargeting using the TCO/Tz cycloaddition mainly focus on two types of radiolabeled tetrazines (*i.e*. [^177^Lu]Lu-Tz-**3** and [^177^Lu]Lu-Tz-**4**), with impressive results in both the imaging and therapy of subcutaneous models of colorectal and pancreatic cancers 12,17,18. Nevertheless, these two types of radioligand differ in terms of the Tz structure and the PEG linker length, and they have never been assessed in the context of disseminated tumors. Because the bis(pyridino)-1,2,4,5-tetrazine motif boasts slightly better kinetic properties than the benzyl-1,2,4,5-tetrazine structure 13,45, we synthesized two new derivatives of Tz-**3** − Tz-**1** and Tz-**2**, bearing PEG_3_ and PEG_7_ linkers, respectively − in order to evaluate the pharmacokinetic profiles of [^177^Lu]Lu-Tz-**1-3** *in vivo* and determine the most suitable probe for the PRIT of peritoneal carcinomatosis. PEGylation is known to improve the pharmacokinetic and pharmacodynamic properties of (macro)molecules and nano-objects (nanoparticles, liposomes, *etc.*) 46. Grafting PEG linkers on (macro)molecules enhances their aqueous solubility, prolongs their circulation times, and can confer resistance to uptake and metabolism in the liver 47. In addition, a comparison of the *in vivo* performance of bis(pyridine)-1,2,4,5-tetrazine-based radioligands bearing different kind of linkers (*i.e*. aliphatic chains and PEG chains) revealed that constructs with lipophilic linkers yielded lower activity concentration in tumors, leading to our interest in adding PEG linkers on Tz moieties 48. As the optimal length of the PEG linker remains unclear, we evaluated the pharmacokinetic influence of linkers of three different lengths (PEG_3_, PEG_7_, and PEG_11_).

To the best of our knowledge, the *in vivo* behavior of [^177^Lu]Lu-Tz-**3** and [^177^Lu]Lu-Tz-**4** has never been assessed following i.p. injection. Our previous study established the efficacy of i.p. injections of Tz for pretargeting with 35A7-TCO in a PC model, as the diffusion of the Tz probe is facilitated by its small size 37. We also hypothesized that the local i.p. injection of radiolabeled Tz probes could induce fewer side effects than a classical i.v. route and enhance the retention of the Tz in the peritoneal cavity close to the disseminated tumors. Consequently, to determine the most effective radiolabeled Tz for the PRIT of PC, we first decided to compare the biodistribution profiles of [^177^Lu]Lu-Tz-**1-3** injected intraperitoneally using SPECT imaging and organ radioactivity counting.

SPECT imaging showed the rapid clearance of [^177^Lu]Lu-Tz-**1-3** within a few hours. This comparison of [^177^Lu]Lu-Tz-**1-3** also revealed that the length of the PEG linker of each Tz-bearing radioligand directly influences its* in vivo* behavior. Indeed, it appeared that the shortest linker (*i.e*. PEG_3_, [^177^Lu]Lu-Tz-**1**) promoted the hepatic, intestinal, and renal elimination of the radiolabeled probe, while the longer linkers (*i.e*. PEG_7_ and PEG_11_, [^177^Lu]Lu-Tz-**2** and [^177^Lu]Lu-Tz-**3**, respectively) promoted exclusive urinary clearance. The retention time of [^177^Lu]Lu-Tz-**1** in the peritoneal cavity was longer than that of both [^177^Lu]Lu-Tz-**2** and [^177^Lu]Lu-Tz**-3**. This could be explained by the higher lipophilicity of [^177^Lu]Lu-Tz-**1**. Finally, the high non-specific background signal of [^177^Lu]Lu-Tz-**1** in elimination organs such as the liver and intestines is a definite drawback for imaging in this setting due to the possible presence of lesions in these tissues.

Moving on toward our PRIT study, we have already demonstrated the efficacy of the i.v. injection of 35A7-TCO immunoconjugates for targeting PC 37. In addition, in biodistribution experiments, we noticed that the injection route of 35A7-TCO (*i.e*. i.v. *vs* i.p.) has no significant influence on the *in vivo* performance of the system, but i.v. injections seem more pertinent in the context of the PRIT of PC. Hence, biodistributions and PRIT were both performed by combining an i.v. injection of 35A7-TCO with an i.p. injection of [^177^Lu]Lu-Tz-**1-4**.

Next, we wondered if the bis(pyridino)-1,2,4,5-tetrazine motif contained in [^177^Lu]Lu-Tz-**1-3** − which was successfully employed by Robillard and co-workers − was the most appropriate Tz core structure for the PRIT of PC 14. The pretargeted biodistribution profiles of [^177^Lu]Lu-Tz-**1-3** in mice bearing PC tumors were compared to that of [^177^Lu]Lu-Tz-**4** (a less sterically hindered Tz) to check whether the tumor uptake would be significantly different with a radioligand boasting a different Tz structure 49. Indeed, [^177^Lu]Lu-Tz-**4** has produced impressive results in two different PRIT studies in subcutaneous murine models of pancreatic or colorectal cancers (16.8 ± 3.9 %IA/g and 21.2 ± 2.9 %IA/g in the tumor at 120 h p.i., respectively) 17,18. Moreover, as the chelator in [^177^Lu]Lu-Tz-**4** has an additional free carboxylic acid arm compared to that in [^177^Lu]Lu-Tz-**1-3**, it could facilitate the improved coordination of lutetium-177. However, our pretargeted biodistribution experiments demonstrated similar pharmacokinetic profiles for [^177^Lu]Lu-Tz-**2** and [^177^Lu]Lu-Tz-**4**. Furthermore, no significant activity was found in the bone marrow with either Tz. This would suggest that regardless of the variant of DOTA used (*i.e*. three or four carboxylic acid moieties), no demetalation occurs *in vivo*, data which are in agreement with that published by Läppchen *et al.* 48.

The biodistribution profiles of [^177^Lu]Lu-Tz-**1-4** administered 24 h after the injection of 35A7-TCO clearly demonstrate the potential of TCO/Tz pretargeting in the context of PC. The highest tumoral activity concentrations were observed with [^177^Lu]Lu-Tz-**2** and [^177^Lu]Lu-Tz**-4** (which both contain a PEG_7_ linker), suggesting a close relationship between *in vivo* performance and the hydrophilicity of the probe. Along these lines, we hypothesize that in the case of i.p. administration, the Tz-based probe should be sufficiently soluble to allow access to the whole peritoneal cavity but so soluble as to result in the overly rapid clearance of the radioligand. [^177^Lu]Lu-Tz-**2** seemed more appropriate for pretargeting than [^177^Lu]Lu-Tz-**1**, which accumulated in the liver and exhibited faster peritoneal clearance. Indeed, [^177^Lu]Lu-Tz-**1** does not appear to be an appropriate radioligand for either pretargeted imaging (due to non-specific uptake in the liver and intestines) or therapy (due to possible hepatic degradation) with 35A7-TCO. Furthermore, the tumoral activity concentration values for pretargeting with [^177^Lu]Lu-Tz-**2** and [^177^Lu]Lu-Tz-**4** were not statistically different. As for [^177^Lu]Lu-Tz-**3**, it was cleared too rapidly and demonstrated poorer *in vivo* performance than [^177^Lu]Lu-Tz-**2**, results which led us to eschew further *in vivo* studies with the radioligand.

The dosimetry calculations from these experiments underscore the utility of pretargeting for decreasing radiation doses to non-targeted organs. Indeed, the radiation dose rates are low for healthy, non-target organs and are in the same range as those described by other teams 50. The calculated dose was higher in the liver for [^177^Lu]Lu-Tz-**1** than for [^177^Lu]Lu-Tz-**2-4** and decreased with increasing PEG linker length, data that is generally in agreement with both the biodistribution and SPECT imaging experiments. With respect to the peritoneal tumors, the dose rate was higher for [^177^Lu]Lu-Tz-**2** (0.59 Gy/MBq) than either [^177^Lu]Lu-Tz-**1** or [^177^Lu]Lu-Tz-**3** (0.25 and 0.18 Gy/MBq, respectively). As a result, [^177^Lu]Lu-Tz-**2** probe was selected for subsequent PRIT experiments.

In the recent publication of Membreno and co-workers on the PRIT of subcutaneous CRC, the authors assessed increasing doses of [^177^Lu]Lu-Tz-**4** (*i.e*. 18.7, 37.0 and 55.5 MBq, i.v. injected) and demonstrated a complete survival response associated with a significant total tumor regression even at the lowest injected dose. In addition, no associated toxicity was observed at the highest injected dose. Because (i) [^177^Lu]Lu-Tz-**2** is administered *via* i.p. injection, (ii) PC is a very aggressive cancer, and (iii) no demonstrations of *in vivo* pretargeting in disseminated tumors has yet been published, we selected the median dose of 40 MBq for this proof-of-concept PRIT experiment. Tumor growth was monitored *in vivo* for 20 days after treatment using bioluminescence imaging and was subsequently assessed *via* the *ex vivo* determination of the PCI 24,43. The significant tumor growth slow-down observed 13 days after the administration of [^177^Lu]Lu-Tz-**2** in the PRIT group demonstrated for the first time the efficacy of click chemistry-based PRIT on disseminated tumors. Those promising results encourage us to further optimize this approach to PRIT by varying the amount of 35A7-TCO injected, changing the dose of radioligand, and performing several injections of [^177^Lu]Lu-Tz-**2** 36,18.

In conclusion, this is the first demonstration of the click chemistry-based pretargeted SPECT and PRIT of disseminated tumors. This investigation was performed using four different Tz-based radioligands, and the best *in vivo* results were obtained with probes containing a PEG linker of intermediate length (*i.e*. PEG_7_ in [^177^Lu]Lu-Tz-**2** and [^177^Lu]Lu-Tz**-4**). Finally, a longitudinal therapy study with 35A7-TCO and [^177^Lu]Lu-Tz-**2** revealed that PRIT is a promising new therapeutic approach for peritoneal carcinomatosis from colorectal origin.

## Supplementary Material

Supplementary figures and tables.

## Figures and Tables

**Scheme 1:**
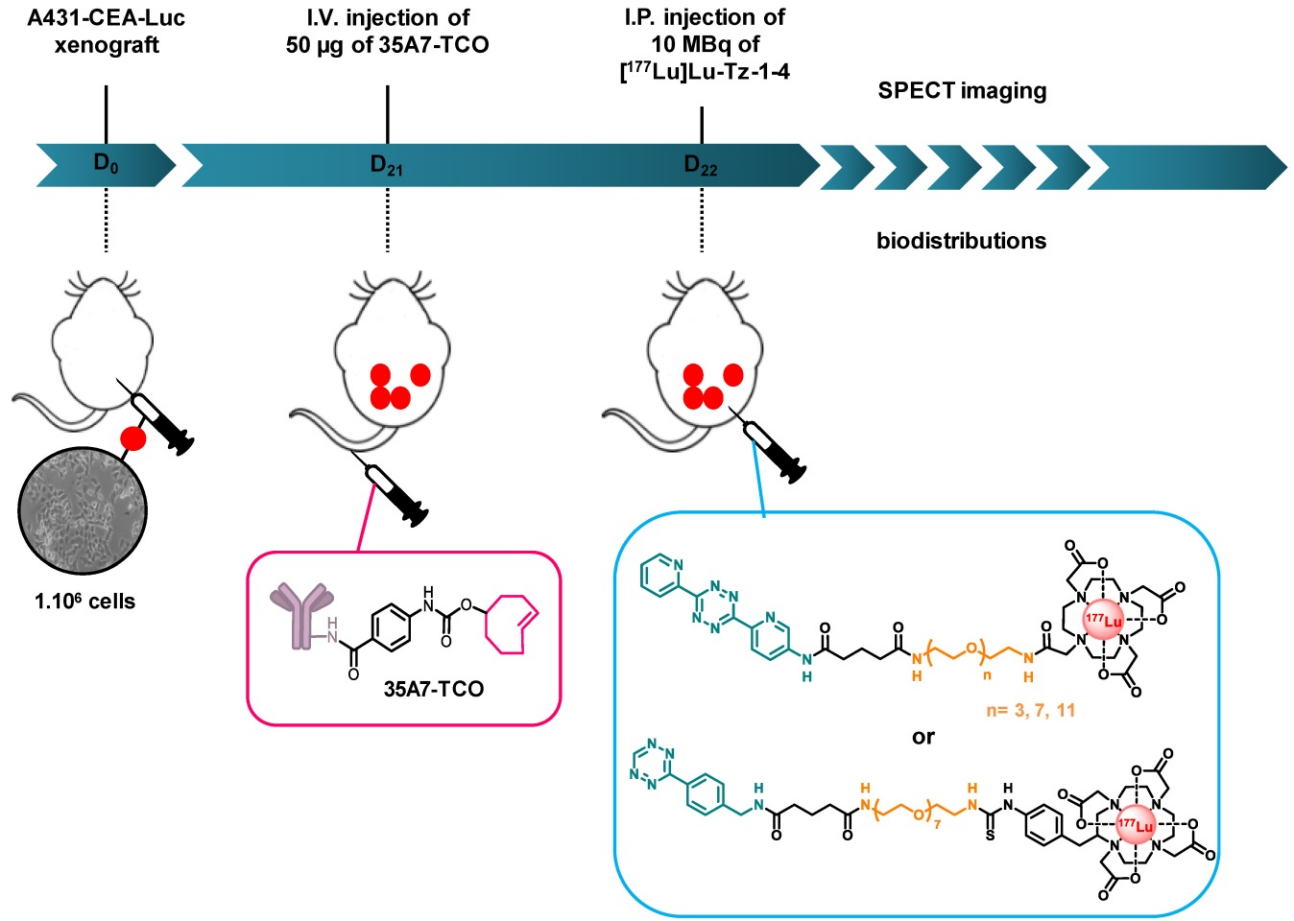
Experimental protocol for determining the biodistribution profiles [^177^Lu]Lu-Tz-**1-4** upon *in vivo* pretargeting with 35A7-TCO. The same protocol was applied to interrogate the biodistribution profiles of [^177^Lu]Lu-Tz-**1-3** alone, except in these cases, the i.p. injections were made at day 21 post-xenograft, as there was no injection of 35A7-TCO.

**Scheme 2:**
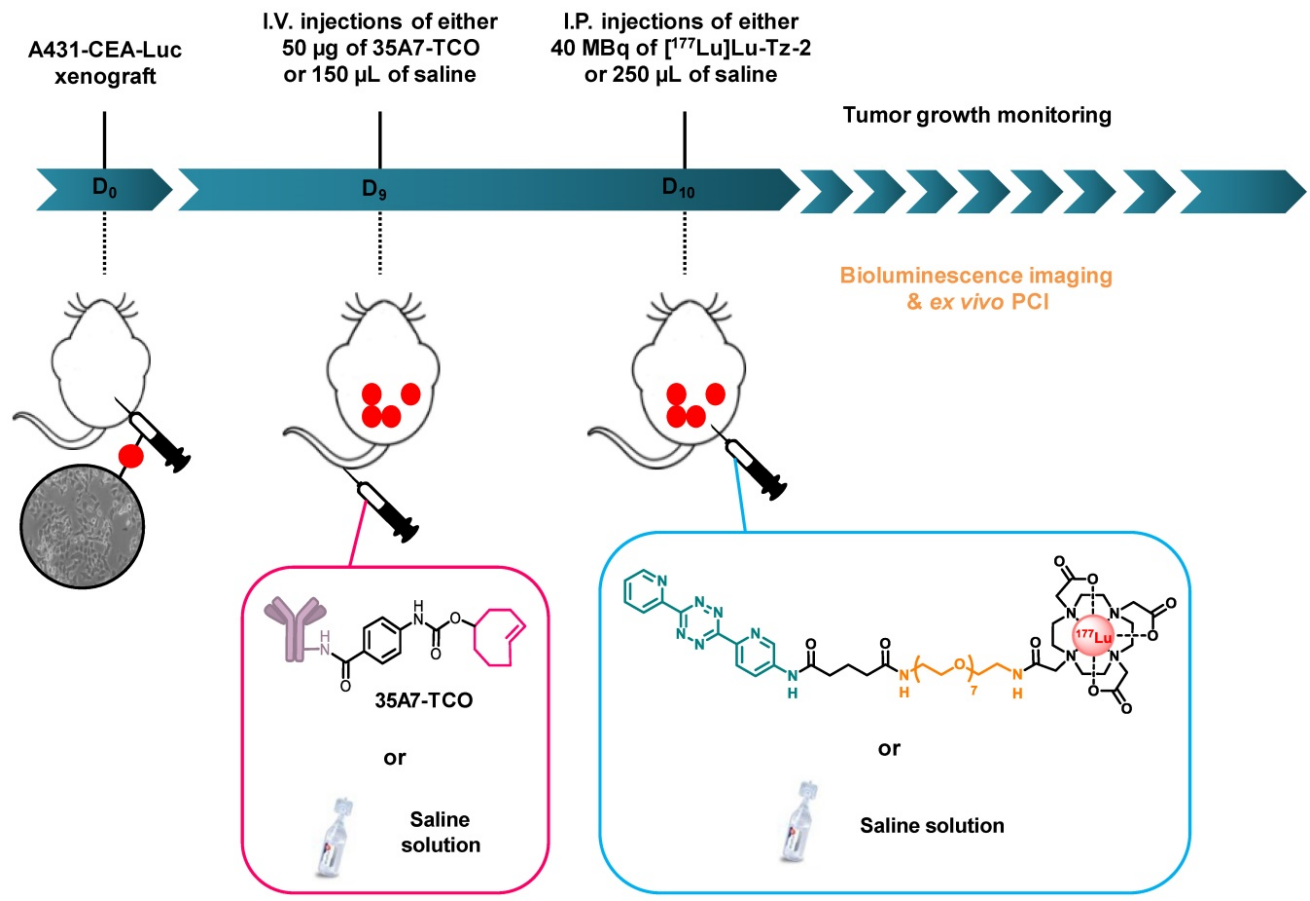
Experimental protocol for the longitudinal PRIT study with 35A7-TCO and [^177^Lu]Lu-Tz-**2**.

**Table 1:**
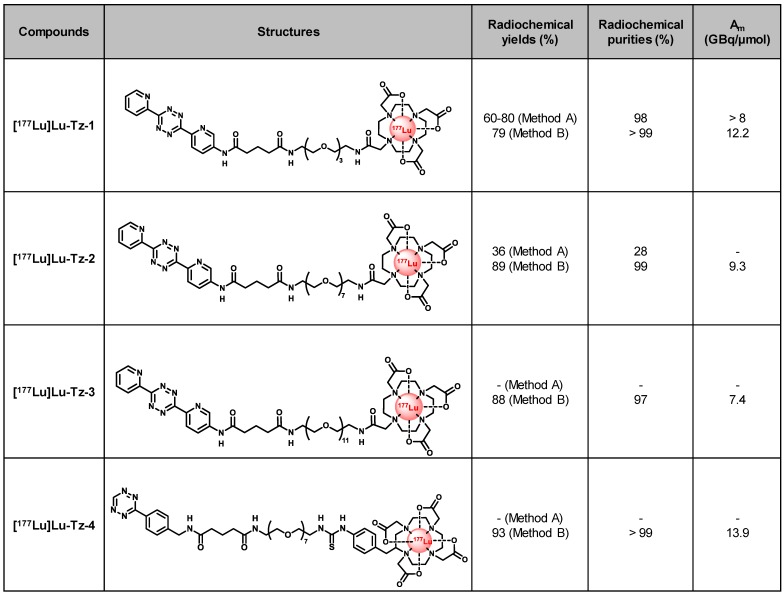
Structures and properties of [^177^Lu]Lu-Tz-**1-4**. A_m_: molar activity.

**Table 2:** Dosimetry data (Mean ± SD) for main organs and peritoneal tumors determined for* in vivo* pretargeting with [^177^Lu]Lu-Tz-**1-4** and 35A7-TCO.

Organs	^177^Lu S-factors (Gy/Bq/s)	Organ Weigth (g)	Dosimetry (Gy/ MBq)							
			[^177^Lu]Lu-Tz-1		[^177^Lu]Lu-Tz-2		[^177^Lu]Lu-Tz-3		[^177^Lu]Lu-Tz-4	
			Mean	SD	Mean	SD	Mean	SD	Mean	SD
Heart	4.66E-11	0.141	1.87E-03	8.74E-04	2.19E-03	7.61E-04	1.00E-03	3.29E-04	3.41E-03	5.38E-04
Lungs	3.78E-11	0.208	4.37E-03	9.67E-04	3.87E-03	1.19E-03	2.31E-03	2.75E-03	6.41E-03	1.32E-03
Liver	1.47E-11	1.333	7.56E-02	1.89E-02	1.60E-02	4.76E-03	1.05E-03	3.50E-04	2.06E-02	1.36E-03
Kidneys	3.95E-11	0.362	2.59E-02	2.52E-03	2.59E-02	2.55E-03	6.62E-03	3.29E-03	2.70E-02	8.25E-04
Spleen	1.62E-10	0.118	2.30E-02	7.34E-03	2.06E-01	2.06E-02	2.14E-02	1.56E-02	2.56E-02	9.75E-03
Brain	5.21E-11	0.388	1.16E-03	9.12E-04	7.95E-04	3.38E-04	8.63E-04	1.10E-03	1.13E-03	4.30E-04
Tumors	1.32E-09	0.016	2.53E-01	1.56E-01	5.92E-01	5.22E-01	1.83E-01	1.13E-01	6.31E-01	5.59E-01

## Abbreviations

%IA/g: percent injected activity *per* gram of tissue
bsAbs: bispecific antibody
CCO: *cis*-cyclooctene
CEA: carcino-embryonic antigen
CRC: colorectal cancer
CT: computed tomography
DOTA: tetracarboxylic acid
HIPEC: hyperthermic intraperitoneal chemotherapy
i.p.: intraperitoneal
i.v.: intravenous
IEDDA: inverse-electron demand Diels-Alder cycloaddition
Lu: lutetium
Luc: luciferase
mAb: monoclonal antibody
MORF: phosphorodiamidate morpholino
PC: peritoneal carcinomatosis
PCI: peritoneal carcinomatosis index
PEG: polyethylene glycol
PET: positron emission tomography
PNA: peptide nucleic acid
PRIT: pretargeted radioimmunotherapy
RIT: radioimmunotherapy
SPECT: single photon emission computed tomography
TCO: *trans*-cyclooctene
Tz: tetrazine
