# Supplementary material for: Pretargeted radioimmunotherapy and SPECT imaging of peritoneal carcinomatosis using bioorthogonal click chemistry: probe selection and first proof-of-concept
Source: Theranostics. 2019 Sep 19;9(22):6706–18. doi: 10.7150/thno.35461 (PMC6771248; doi:10.7150/thno.35461)
Supplement: Supplementary file 1 — Supplementary figures and tables. [file thnov09p6706s1.pdf]

**Supporting information**

# Pretargeted radioimmunotherapy and SPECT imaging of peritoneal carcinomatosis using bioorthogonal click chemistry: probe selection and first proof-of-concept

Aurélie Rondon[1,2], Sébastien Schmitt[1], Arnaud Briat[1], Nancy Ty[1], Lydia Maigne[3], Mercedes Quintana[1], Rosemery Membreno[4], Brian Zeglis[4,5], Isabelle Navarro-Teulon[2], Jean-Pierre Pouget[2], Jean-Michel Chezal[1], Elisabeth Miot-Noirault[1], Emmanuel Moreau[1¤] and Françoise Degoul[1*¤]

[1] Université Clermont-Auvergne, Imagerie Moléculaire et Stratégies Théranostiques, BP 184, F-63005 Clermont-Ferrand, France. Inserm, U 1240, F-63000 Clermont-Ferrand, France. Centre Jean Perrin, F-63011 Clermont-Ferrand, France.

[2] Institut de Recherche en Cancérologie (IRCM), U1194 – Université Montpellier – ICM, Radiobiology and Targeted Radiotherapy, 34298 Montpellier cedex 5.

[3]Laboratoire de Physique de Clermont, UMR 6533 CNRS/IN2P3, Université Clermont Auvergne, 63178 - Aubière Cedex

[4]Department of Chemistry, Hunter College, City University of New York, New York, NY, USA 10028
[5]Department of Radiology, Memorial Sloan Kettering Cancer Center, New York, NY, USA 10028

*** Corresponding Author:**

Françoise DEGOUL
IMoST, UMR 1240, INSERM, UCA
58 Rue Montalembert
630005 Clermont-Ferrand
francoise.degoul@inserm.fr

## 1. Supplementary material and methods

### 1.1. Material for chemical syntheses

Unless otherwise mentioned, all manipulations were performed under argon atmosphere; all reagents were purchased from the following commercial suppliers: Sigma-Aldrich, Acros Organics, Carlo Erba, TCI Europa, Alpha Aesar. DOTA-NHS was purchased from Chematech (Dijon, France). Anhydrous DMF, anhydrous trimethylamine, anhydrous pyridine were purchased from Acros Organics. THF was dried over a Pure Solv™ Micro Solvent Purification System (Sigma-Aldrich) with an alumina column. Dichloromethane was distilled over calcium hydride. Reactions were monitored by thin layer chromatography (TLC) on silica gel (60 F254; Alugram XTra G/UV254; Macherey-Nagel) or alumina gel (Alumina oxide 60A + F254 neutral; Macherey-Nagel) and visualized with UV light (UV lamp Fisher Bioblock Scientific, 365 nm or 254 nm). Purifications by flash column chromatography were performed on silica gel (Chromagel 60 ACC, 40-63 µm, Carlo Erba Reagents). Purifications on RP18 were conducted on a Combiflash *EZ* prep system (Teledyne Isco) with a RedispSeP C18 column (250 mm x 20 mm, pores 100 Å, particles 5 µm). Sep-Pak C18 light cartridges were purchased from Waters (Milford, MA, USA). Uncorrected melting points (mp) were measured on an IA9100 Digital Melting Point Apparatus. Infrared spectra (IR) were recorded in the range 4000-440 $cm^{-1}$ on a Nicolet IS10 with attenuated total reflectance (ATR) accessory. Nuclear magnetic resonance (NMR) spectra were acquired on Bruker AC-200 or 400 operating at 200 or 400 MHz for $^1H$ NMR and 50 or 100 MHz for $^{13}C$ NMR, respectively. All $^1H$ and $^{13}C$ NMR spectra are reported in δ units, parts per million (ppm). Coupling constants were indicated in Hertz (Hz). The following abbreviations are used for spin multiplicity: s = singlet, d = doublet, t = triplet, quint = quintuplet, m =multiplet, and brs = broad singlet. High resolution mass spectra (HRMS) were recorded on an Alliance 2695 (Waters) liquid chromatography coupled with a Q-ToF micro (Waters/Micromass) spectrometer (UCA-START, Clermont-Ferrand, France).

### 1.2. Access to $PEG_4$, $PEG_8$ and $PEG_{12}$ aminolinkers

#### 1.2.1. $PEG_4$ derivatives

Scheme S1[a]

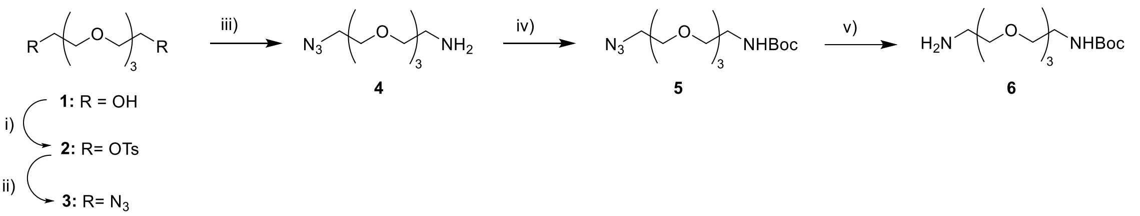


[a]**Reagents and conditions:** i) TsCl, $CH_2Cl_2$, rt, 16h, 90%; ii) $NaN_3$, sol sat. $NaHCO_3$, reflux, 20h, 96%; iii) $PPh_3$, diethyl ether, $H_3PO_4$ (0.65M), rt, 20h, 46%; iv) $Boc_2O$, $CH_2Cl_2$,DMAP, rt, 24 h, 86%; v) $H_2$, Pd/C 10%, rt, 20 h, 90%.

N.B.: Before all reactions, PEGylated compounds were dried twice by co-evaporation with dry toluene (2x40 mL) under reduce pressure.

**1.2.1.1. ((Oxybis(ethane-2,1-diyl))bis(oxy))bis(ethane-2,1-diyl) bis(4-methylbenzenesulfonate) (2)**[1]

To a solution of (**1**) (18.3 g, 94.21 mmol) in dry freshly distilled $CH_2Cl_2$ (120 mL), cooled to 0 °C, TsCl (188.44 mmol) was added portionwise, followed by DMAP (4.71 mmol). After stirring at 0 °C for 10 min, $NEt_3$ (94.21 mmol) was added dropwise. The resulting mixture wad stirred at room temperature

[1] M.E. Bakleh, V. Sol, K. Estieu-Gionnet, R. Granet, G. Déléris, P. Krausz. An efficient route to VEGF-like peptide porphyrin conjugates via microwave-assisted 'click-chemistry'. *Tetrahedron* **2009**, *65*, 7385-7392.

for 16 h. Water (80 mL) was added and the resulting solution was extracted with $CH_2Cl_2$ (3 × 50 mL). The combined organic layers were washed with brine (50 mL), dried over $MgSO_4$, filtered and evaporated under reduced pressure. The crude product was purified by column chromatography on silica gel eluted with ethyl acetate to afford compound **(2)** (90%). $^1$H NMR (400 MHz, $CDCl_3$) $\delta$ 2.39 (s, 6H), 3.54-3.64 (m, 12H), 4.09-4.12 (m, 4H), 7.31 (d, 4H, J = 8.5 Hz), 7.75 (d, 4H, J = 8.5 Hz). These data are in agreement with those of the literature.[1]

**1.2.1.2. 1-Azido-2-(2-(2-(2-azidoethoxy)ethoxy)ethoxy)ethane (3)**[1]

Compound (**2**) (40 g, 79.58 mmol) was dissolved in a saturated aqueous solution of $NaHCO_3$ (150 mL) and $NaN_3$ (19.10 mmol) was added. The resulting mixture was stirred at reflux for 20 h. After cooling to RT, the mixture was extracted with $CH_2Cl_2$ (3×50 mL), washed with water (40 mL), brine (40 mL), dried over $MgSO_4$, filtered and evaporated under reduced pressure. The crude product was purified by column chromatography on silica gel eluted with ethyl acetate/cyclohexane (5/5, v/v) to afford compound (**3**) (96%). $^1$H NMR (200 MHz, $CDCl_3$) $\delta$ 3.24-3.26 (m, 4H), 3.46-3.58 (m, 12H). These data are in agreement with those of the literature.[1]

**1.2.1.3. 2-(2-(2-(2-Azidoethoxy)ethoxy)ethoxy)ethan-1-amine (4)**[1]

To a solution of compound (**3**) (4.28 g, 17.52 mmol) in a 0.65 M aqueous solution of $H_3PO_4$ (23 mL) at RT was added dropwise a solution of $PPh_3$ (15.25 mmol) in diethyl ether (20 mL). The resulting mixture was stirred for 20 h. The reaction mixture was extracted with diethyl ether (3×30 mL). KOH (59.05 mmol) was added to the aqueous layer. Traces of ether were evaporated and the aqueous solution was cooled to 4 °C overnight. The precipitate was filtered off. Then, KOH (165.23 mmol) was added to the filtrate and the solution was extracted with diethyl ether (3×60 mL). The combined organic layer were washed with brine (50 mL), dried over $MgSO_4$, filtered and evaporated under reduced pressure. The crude product was dissolved in $CH_2Cl_2$ (80 mL) and the solution was washed with 0.65 M $H_3PO_4$ (3×50 mL). The combined aqueous solutions were basified with KOH until pH 14, then were extracted with $CH_2Cl_2$ (3×60 mL). The combined organic layers were dried over $MgSO_4$, filtered and evaporated under reduced pressure to afford compound (**4**) (46%) which was used without further purification in the next step. $^1$H NMR (200 MHz, $CDCl_3$) $\delta$ 2.75 (brs, 2H), 3.26-3.31 (m, 2H), 3.39-3.62 (m, 12H). These data are in agreement with those of the literature.[1]

**1.2.1.4. *tert*-Butyl (2-(2-(2-(2-azidoethoxy)ethoxy)ethoxy)ethyl)carbamate (5)**[2]

To a solution of compound (**4**) (2 g, 9.16 mmol) in $CH_2Cl_2$ (85 mL) was added $Boc_2O$ (10.99 mmol) portionwise. The reaction mixture was stirred at RT for 24 h, then evaporated under reduced pressure. The crude product was purified by column chromatography on silica gel eluted with ethyl acetate/$CH_2Cl_2$/EtOH (70/5/25, v/v/v) to afford compound (**5**) (86%). $^1$H NMR (200 MHz, $CDCl_3$) $\delta$ 1.41 (s, 9H), 3.27-3.39 (m, 4H), 3.49-3.51 (m, 2H), 3.58-3.68 (m, 10H), 5.06 (brs, 1H). These data are in agreement with those of the literature.[2]

[2] J. Davila, A. Chassepot, J. Longo, F. Boulmedais, A. Reisch, B. Frisch, F. Meyer, J-C. Voegel, P. J. Mésini, Bernard Senger, M-H. Metz-Boutigue, J. Hemmerlé, P. Lavalle, P. Schaaf, and L. Jierry. Cyto-mechanoresponsive Polyelectrolyte Multilayer Films. *J. Am. Chem. Soc.*, **2012**, *134*, 1, 83-86.

#### 1.2.1.5. *tert*-Butyl (2-(2-(2-(2-aminoethoxy)ethoxy)ethoxy)ethyl)carbamate (6)[3]

To a solution of compound (5) (3 g, 94.2 mmol) in absolute EtOH (120 mL) was added 10% Pd/C (0.3 g) portionwise. The resulting mixture was degassed three times and was stirred under dihydrogen atmosphere (1 bar) for 20 h. The resulting mixture was filtered through a pad of Celite® 545 and the filtrate was evaporated under reduced pressure to dryness to afford compound (**6**) which was used in the next step without further purification (90%). $^1$H NMR (200 MHz, $CDCl_3$) δ 1.42 (s, 9H), 2.37 (brs, 2H), 2.79-2.87 (m, 2H), 3.27-3.30 (m, 2H), 3.48-3.72 (m, 10H), 5.21 (brs, 1H). These data are in agreement with those of the literature.[3]

### 1.2.2. $PEG_8$ and $PEG_{12}$ derivatives

**Scheme S2**[a]

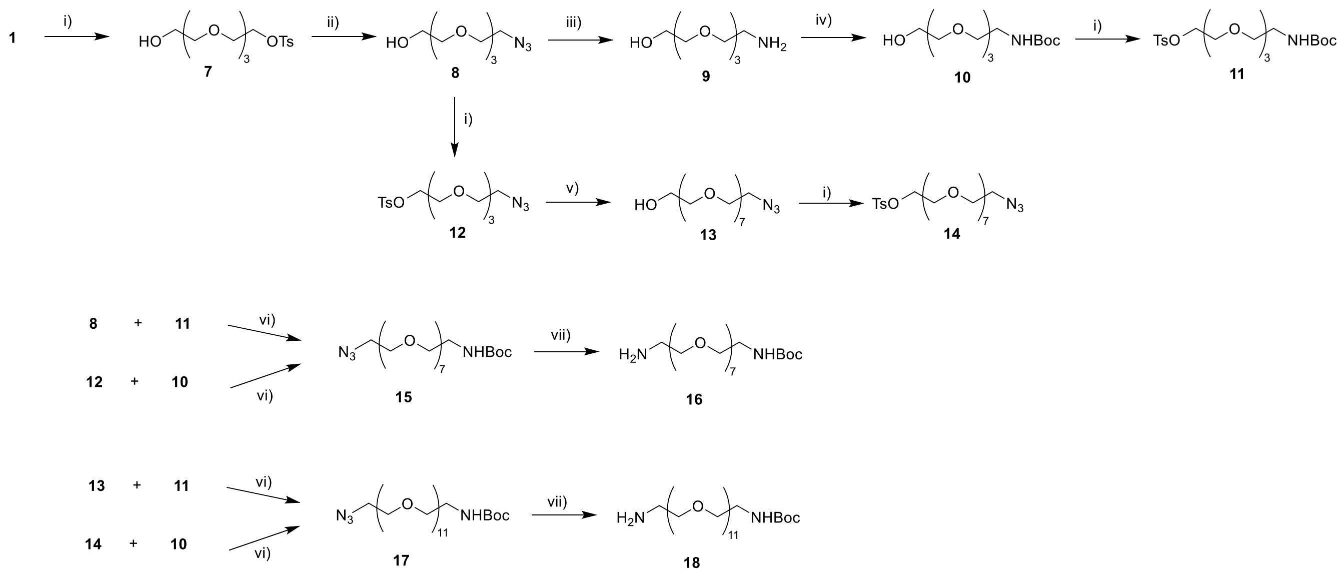

**[a]Reagents and conditions:** i) TsCl, $NEt_3$, DMAP, $CH_2Cl_2$, rt, 16 h; ii) $NaN_3$, saturated solution of $NaHCO_3$, reflux, 24 h; iii) $H_2$, Pd/C 10%, rt, 20 h; iv) $Boc_2O$, $CH_2Cl_2$, DMAP, rt, 20 h; v) (**1**), NaH, THF, 0°C then reflux; (vi) NaH, THF, 0°C then reflux; vii) $PPh_3$, EtOH, THF, $NH_4OH$, rt.

#### 1.2.2.1. 2-(2-(2-(2-Hydroxyethoxy)ethoxy)ethoxy)ethyl 4-methylbenzenesulfonate (7)[4]

To a solution of compound (**1**) (18.3 g, 94.21 mmol) in dry freshly distilled $CH_2Cl_2$ (120 mL), cooled to 0 °C, was added TsCl (56.53 mmol) portionwise, followed by $NEt_3$ (70.66 mmol) and DMAP (4.71 mmol) under argon atmosphere. After stirring at 0 °C for 10 min, the resulting mixture was stirred at RT for 16 h. Water (80 mL) was added and the resulting solution was extracted with $CH_2Cl_2$ (3×50 mL). The combined organic layers were washed with brine (50 mL), dried over $MgSO_4$, filtered and evaporated under reduced pressure. The crude product was purified by column chromatography on silica gel eluted with ethyl acetate/cyclohexane (3/7, v/v) to afford the ditosylated compound (11 %), followed by elution with pure ethyl acetate to afford the expected compound (**7**) (74%). $^1$H NMR (200 MHz, $CDCl_3$) δ 2.41 (s, 3H), 3.56-3.73 (m, 14H), 4.10-4.15 (m, 2H), 7.31 (d, 2H, J = 8.0 Hz), 7.76 (d, 2H, J = 8.0 Hz). These data are in agreement with those of the literature.[4]

---

[3] W. Zhang, D. T. Nowlan, L. M. Thomson, W. M. Lackowski and E. E. Simanek. Orthogonal, Convergent Syntheses of Dendrimers Based on Melamine with One or Two Unique Surface Sites for Manipulation. *J. Am. Chem. Soc.*, **2001**, *123*, 37, 8914-8922.

[4] C. Steinem Andreas, J. Karstenvon dem Bruch, K. Reihs, J. Goossens and H-J, Galla. Valinomycin-mediated transport of alkali cations through solid supported membranes. *Bioelectrochem. Bioenerg.* **1998**, *45*, 1, 17-26.

**1.2.2.2. 2-(2-(2-(2-Azidoethoxy)ethoxy)ethoxy)ethan-1-ol (8)[5]**

To a solution of compound (**7**) (5.62 g, 16.13 mmol) in a saturated aqueous solution of $NaHCO_3$ (50 mL), $NaN_3$ (24.19 mmol) was added. The resulting mixture was stirred at reflux for 24 h. After cooling to RT, the mixture was extracted with $CH_2Cl_2$ (3×50 mL), washed with water (40 mL), brine (40 mL), dried over $MgSO_4$, filtered and evaporated under reduced pressure. The crude product was purified by column chromatography on silica gel eluted with ethyl acetate to afford compound (**8**) (81%). $^1H$ NMR (200 MHz, $CDCl_3$) $\delta$ 3.02 (brs, 1H), 3.50-3.53 (m, 2H), 3.55-3.62 (m, 14H). These data are in agreement with those of the literature.[5]

**1.2.2.3. 2-(2-(2-(2-Aminoethoxy)ethoxy)ethoxy)ethan-1-ol (9)[6]**

To a solution of compound (**8**) (8.57 g, 4.30 mmol) in absolute EtOH (120 mL) was added 10% Pd/C (0.86 g) portionwise. The resulting mixture was degassed three times and was stirred under dihydrogen atmosphere (1 bar) for 20 h. The reaction solution was filtered through a pad of Celite® 545 and the filtrate was evaporated under reduced pressure to dryness to afford compound (**9**) which was used in the next step without further purification (82%). $^1H$ NMR (200 MHz, $CDCl_3$) $\delta$ 2.88-2.96 (m, 5H), 3.55-3.78 (m, 14H). These data are in agreement with those of the literature.[6]

**1.2.2.3. *tert*-Butyl (2-(2-(2-(2-hydroxyethoxy)ethoxy)ethoxy)ethyl)carbamate (10)[7]**

To a solution of compound (**9**) (3.62 g, 18.73 mmol) in $CH_2Cl_2$ (75 mL) was added $Boc_2O$ (18.73 mmol). The resulting mixture was stirred at RT for 20 h, then was evaporated under reduced pressure. The crude product was purified by column chromatography on silica gel eluted with ethyl acetate/$CH_2Cl_2$/EtOH (70/5/25, v/v/v) to afford compound (**10**) (78%). $^1H$ NMR (200 MHz, $CDCl_3$) $\delta$ 1.41 (s, 9H), 3.27-3.39 (m, 2H), 3.48-3.59 (m, 2H), 3.61-3.73 (m, 12H), 5.58 (brs, 1H). These data are in agreement with those of the literature.[7]

**1.2.2.4. 2,2-Dimethyl-4-oxo-3,8,11,14-tetraoxa-5-azahexadecan-16-yl 4-methylbenzenesulfonate (11)[8]**

To a solution of compound (**10**) (2.80 g, 9.56 mmol) in $CH_2Cl_2$ (80 mL), cooled to 0 °C, were successively added TsCl (10.52 mmol), DMAP (0.48 mmol) and $NEt_3$ (21.99 mmol). The reaction mixture was stirred at RT for 40 h. A saturated aqueous solution of $NaHCO_3$ was added (30 mL) and the resulting mixture was extracted with dichloromethane (3×20 mL). The combined organic layers were dried over $MgSO_4$, filtered and evaporated under reduced pressure. The crude product was purified by column chromatography on silica gel eluted with $CH_2Cl_2$/EtOH (95/5, v/v) to afford compound (**11**) (67%). $^1H$ NMR (200 MHz, $CDCl_3$) $\delta$ 1.45 (s, 9H), 2.45 (s, 3H), 3.27-3.32 (m, 2H), 3.51-3.73 (m, 12H), 4.15-4.19 (m, 2H), 5.04 (brs, 1H), 7.34 (d, 2H, $J$ = 8.4 Hz), 7.81 (d, 2H, $J$ = 8.4 Hz). These data are in agreement with those of the literature.[8]

[5] C. R. Bertozzi and M. D. Bednarski. The synthesis of heterobifunctional linkers for the conjugation of ligands to molecular probes. *J. Org. Chem.*, **1991**, *56*, 13, 4326-4329.

[6] P. Besenius, P. A. G. Cormack, R. F. Ludlow, S. Otto and D. C. Sherrington. Polymer-supported cationic templates for molecular recognition of anionic hosts in water. *Chem. Comm.*, **2008**, *24*, 2809-2811.

[7] J. K. Pokorski, K. Breitenkamp, L. O. Liepold, S. Qazi, M. G. Finn. Functional virus-based polymer-protein nanoparticles by atom transfer radical polymerization. *J. Am. Chem. Soc.* **2011**, *133*, 24, 9242-9245.

[8] K. S. Yang, G. Budin, C. Tassa, O. Kister and R. Weissleder. Bioorthogonal approach to identify unsuspected drug targets in live cells. *Angew. Chem. Int. Ed.*, **2013**, *52*, 40, 10593-10597.

**1.2.2.7. 2-(2-(2-(2-Azidoethoxy)ethoxy)ethoxy)ethyl 4-methylbenzenesulfonate (12)**[9]

To a solution of compound (**8**) (4.0 g, 18.24 mmol) in $CH_2Cl_2$, cooled to 0 °C, were successively added DMAP (1.36 mmol), TsCl (27.36 mmol) and $NEt_3$ (13.68 mmol). The resulting mixture was stirred at reflux for 12 h. After cooling to room temperature, the mixture was poured into water (40 mL), and the organic layer was separated, dried over $MgSO_4$ and evaporated under reduced pressure. The crude product was purified by column chromatography on silica gel eluted with ethyl acetate/cyclohexane (5/5, v/v) to afford compound (**12**) (75%). $^1H$ NMR (200 MHz, $CDCl_3$) $\delta$ 2.36 (s, 3H), 3.27-3.32 (m, 2H), 3.30-3.32 (m, 2H), 3.50-3.59 (m, 12H), 4.04-4.09 (m, 2H), 7.27 (d, *J* = 8.3 Hz, 2H), 7.71 (d, *J* = 8.3 Hz, 2H). These data are in agreement with those of the literature.[9]

**1.2.2.5. 23-Azido-3,6,9,12,15,18,21-heptaoxatricosan-1-ol (13)**[10]

To a solution of compound (**1**) (8.21 g, 21.98 mmol) in dry THF, cooled 0 °C, was added NaH (60 % w/w in mineral oil, 10.99 mmol) portionwise. After stirring at 0 °C for 1 h, a solution of compound (**12**) (21.98 mmol) dissolved in dry freshly distilled THF (10 mL) was added dropwise at 0 °C. The resulting mixture was heated at reflux for 16 h. After cooling to RT, water (40 mL) was added to the reaction mixture and the resulting solution was extracted with ethyl acetate (3×40 mL). The combined organic layers were washed with brine (50 mL), dried over $MgSO_4$, filtered and evaporated under reduced pressure. The crude product was purified by column chromatography on silica gel eluted with $CH_2Cl_2$/EtOH (95/5, v/v) to afford compound (**13**) (71%). $^1H$ NMR (200 MHz, $CDCl_3$) $\delta$ 3.06 (brs, 1H), 3.34-3.39 (m, 2H), 3.55-3.71 (m, 30H). These data are in agreement with those of the literature.[10]

**1.2.2.6. 23-Azido-3,6,9,12,15,18,21-heptaoxatricosyl 4-methylbenzenesulfonate (14)**[11]

To a solution of compound (**13**) (8.76 g, 15.93 mmol) in $CH_2Cl_2$ cooled to 0 °C were successively added DMAP (0.79 mmol), TsCl (19.12 mmol) and $NEt_3$ (19.12 mmol). The resulting mixture was stirred at reflux for 18 h. After cooling to room temperature, the mixture was poured into water (40 mL), and the organic layer was separated, dried over $MgSO_4$ and evaporated under reduced pressure. The crude product was purified by column chromatography on silica gel eluted with ethyl acetate/cyclohexane (5/5, v/v) to afford compound (**14**) (81%). $^1H$ NMR (400 MHz, $CDCl_3$) $\delta$ 2.45 (s, 3H), 3.39 (t, *J* = 5.0 Hz, 2H), 3.52-3.75 (m, 28H) 4.16 (t, *J* = 4.9 Hz, 2H), 7.34 (d, *J* = 8,3 Hz, 2H), 7.80 (d, *J* = 8,3 Hz, 2H). These data are in agreement with those of the literature.[11]

**1.2.2.8. *tert*-Butyl (23-azido-3,6,9,12,15,18,21-heptaoxatricosyl)carbamate (15)**

<u>*Method A:*</u> To a solution of compound (**8**) (0.50 g, 1.92 mmol) in dry THF, cooled to 0 °C, NaH (60% w/w in mineral oil, 5.75 mmol) was added portionwise. After stirring at 0 °C for 1 h, a solution of compound (**11**) (1.74 mmol) dissolved in dry THF (10 mL) was added dropwise, followed by DMAP (0.19 mmol). The resulting mixture was stirred at RT for 16 h. A solution of water (30 mL) was added and the resulting

[9] B. C. Sanders, F. Friscourt, P. A. Ledin, N.E. Mbua, S. Arumugam, J. Guo, T. J. Boltje, V. V. Popik, G.J. Boons. Metal-free sequential [3+2]-dipolar cycloadditions using cyclooctynes and 1,3-dipoles of different reactivity. *J Am Chem Soc.* **2011**, *133*, 4, 949-957.

[10] C. Thauvin, A. Perino, E. Contal, E. Morin, P. Schultz, S. Meunier and A. Wagner. Programmed Dispersions of MWNTs in Aqueous Media by Coating with Photopolymerizable Synthetic Amphiphiles. *J. Phys. Chem. C*, **2011**, *115*, 15, 7319-7322.

[11] H. S. Gill, J. N. Tinianow, A. Ogasawara, J. E. Flores, A. N. Vanderbilt, H. Raab, J. M. Scheer, R. Vandlen, S.-P. Williams and J. Marik. A Modular Platform for the Rapid Site-Specific Radiolabeling of Proteins with 18F Exemplified by Quantitative Positron Emission Tomography of Human Epidermal Growth Factor Receptor 2. *J. Med. Chem.*, **2009**, *52*, 19, 5816-5825.

solution was extracted with ethyl acetate (3×30 mL). The combined organic layers were washed with brine (50 mL), dried over $MgSO_4$, filtered and evaporated under reduced pressure. The crude product was purified by column chromatography on silica gel eluted with ethyl acetate/$CH_2Cl_2$/EtOH (70/25/5, v/v/v) to afford compound (**15**) (68%). $^1$H NMR (200 MHz, $CDCl_3$) δ 1.43 (s, 9H), 3.26-3.51 (m, 4H), 3.53-3.75 (m, 28H), 5.12 (brs, 1H); NMR $^{13}$C (125 MHz, $CDCl_3$) δ 28.3, 50.5, 69.8, 69.9, 70.0, 70.3, 163.0; HRMS for $C_{21}H_{43}N_4O_9$ m/z $[M+H]^+$ calc.: 495.3014; found: 495.3030.

*Method B:* To a solution of compound (**10**) (0.125 g, 0.43 mmol) in dry THF cooled to 0 °C, NaH (60 % w/w in mineral oil, 1.28 mmol) was added portionwise. After stirring at 0 °C for 1 h, a solution of compound (**12**) (0.44 mmol) dissolved in dry THF (10 mL) was added dropwise. The resulting mixture was stirred at RT for 16 h. A saturated aqueous solution of $NaHCO_3$ (30 mL) was added and the resulting solution was extracted with ethyl acetate (3×30 mL). The combined organic layers were washed with brine (50 mL), dried over $MgSO_4$, filtered and evaporated under reduced pressure. The crude product was purified by column chromatography on silica gel eluted with ethyl acetate/$CH_2Cl_2$/EtOH (70/25/5, v/v/v) to afford compound (**15**) (37%). $^1$H NMR (200 MHz, $CDCl_3$) δ 1.43 (s, 9H), 3.26-3.51 (m, 4H), 3.53-3.75 (m, 28H), 5.12 (brs, 1H).

### 1.2.2.9. *tert*-Butyl (23-amino-3,6,9,12,15,18,21-heptaoxatricosyl)carbamate (16)[12]

To a solution of compound (**15**) (0.15 g, 0.30 mmol) in a mixture of absolute EtOH and THF (1.8 mL, 2/1, v/v), $PPh_3$ (0.64 mmol) was added portionwise, followed by a 25% aqueous solution of $NH_4OH$ (50 µL). The resulting mixture was stirred at RT for 14 h. The reaction mixture was evaporated under reduced pressure. The crude product was dissolved in water (5 mL), and then extracted with $CH_2Cl_2$ (3×5 mL). The combined organic layers were dried over $MgSO_4$, filtered and evaporated under reduced pressure. The crude product was purified on a short pad of silica gel eluted with ethyl acetate/EtOH (90/10, v/v), followed by ethyl acetate/$CH_2Cl_2$/EtOH/25% aq. $NH_4OH$ (75/25/5/0.2, v/v/v/v) to afford compound (**16**) (87%). $^1$H NMR (200 MHz, $CDCl_3$) δ 1.43 (s, 9H), 2.96-2.98 (m, 2H), 3.29-3.31 (m, 2H), 3.54-3.76 (m, 32H), 5.32 (brs, 1H). These data are in agreement with those of the literature.[12]

### 1.2.2.10. *tert*-Butyl (35-azido-3,6,9,12,15,18,21,24,27,30,33-undecaoxapentatriacontyl)carbamate (17)

*Method A:* To a solution of compound (**13**) (0.75 g, 1.90 mmol) in dry THF cooled to 0 °C, NaH (60% w/w in mineral oil, 5.69 mmol) was added portionwise. After stirring at 0 °C for 1 h, a solution of compound (**11**) (1.88 mmol) dissolved in dry THF (10 mL) was added dropwise, followed by DMAP (0.19 mmol). The resulting mixture was stirred at RT for 20 h. Water (30 mL) was added and the resulting solution was extracted with ethyl acetate (3×30 mL). The combined organic layers were washed with brine (50 mL), dried over $MgSO_4$, filtered and evaporated under reduced pressure. The crude product was purified by column chromatography on silica gel eluted with ethyl acetate/$CH_2Cl_2$/EtOH (70/25/5, v/v/v) to afford compound (**17**) (63%). $^1$H NMR (200 MHz, $CDCl_3$) δ 1.43 (s, 9H), 3.18-3.89 (m, 48H), 5.14 (brs, 1H); NMR $^{13}$C (125 MHz, $CDCl_3$) δ 28.3, 40.5, 52.5, 69.8, 69.9, 71.0, 80.3, 162.2; HRMS for $C_{29}H_{58}N_4O_{13}$ m/z $[M+H]^+$ calc.: 671.4084; found: 671.4073.

*Method B:* To a solution of compound (**10**) (0.125 g, 0.43 mmol) in dry THF, cooled to 0 °C, NaH (60% w/w in mineral oil, 1.28 mmol) was added portionwise. After stirring at 0 °C for 1 h, a solution of

[12] M. Hashimoto, J. Yang and G. D. Holman. Cell-surface recognition of biotinylated membrane proteins requires very long spacer arms: an example from glucose-transporter probes. *ChemBioChem*, **2001**, *22*, 1, 52-59.

compound (**14**) (0.44 mmol) dissolved in dry THF (10 mL) was added dropwise. The resulting mixture was stirred at RT for 16 h. A saturated aqueous solution of $NaHCO_3$ (30 mL) was added and the resulting solution was extracted with ethyl acetate (3×30 mL). The combined organic layers were washed with brine (50 mL), dried over $MgSO_4$, filtered and evaporated under reduced pressure. The crude product was purified on silica gel eluted with ethyl acetate/$CH_2Cl_2$/EtOH (70/25/5, v/v/v) to afford compound (**17**) (17%). $^1$H NMR (200 MHz, $CDCl_3$) $\delta$ 1.43 (s, 9H), 3.18-3.89 (m, 48H), 5.14 (brs, 1H).

#### 1.2.2.11. *tert*-Butyl (35-amino-3,6,9,12,15,18,21,24,27,30,33-undecaoxapentatriacontyl)carbamate (18)

To a solution of compound (**17**) (0.2 g, 0.30 mmol) in a mixture of absolute EtOH and THF (1.8 mL, 2/1, v/v), $PPh_3$ (0.63 mmol) was added portionwise, followed by 25% aq. $NH_4OH$ (50 µL). The resulting mixture was stirred at RT for 14 h. The reaction mixture was evaporated under reduced pressure. The crude product was dissolved in water (5 mL), then extracted with $CH_2Cl_2$ (3×5 mL). The combined organic layers were dried over $MgSO_4$, filtered and evaporated under reduced pressure. The crude product was purified on a short pad of silica gel eluted with ethyl acetate/EtOH (90/10, v/v), followed by ethyl acetate/$CH_2Cl_2$/EtOH/25% $NH_4OH$ (75/25/5/0.2, v/v/v/v) to afford compound (**18**) (54%). $^1$H NMR (200 MHz, $CDCl_3$) $\delta$ 1.46 (s, 9H), 2.70 (m, 2H), 2.94-2.97 (m, 2H), 3.31-3.33 (m, 2H), 3.54-3.76 (m, 42H), 5.13 (brs, 1H); $^{13}$C NMR (125 MHz, $CDCl_3$) $\delta$ 28.3, 39.7, 40.0, 61.6, 66.4, 70.5, 72.7, 78.6, 156.5; HRMS for $C_{21}H_{44}N_2O_9$ m/z $[M+H]^+$ calc.: 469.3116; found: 169.3119.

## 1.2.3. Access to $TzPEG_nDOTA$

**Scheme S3**[a]

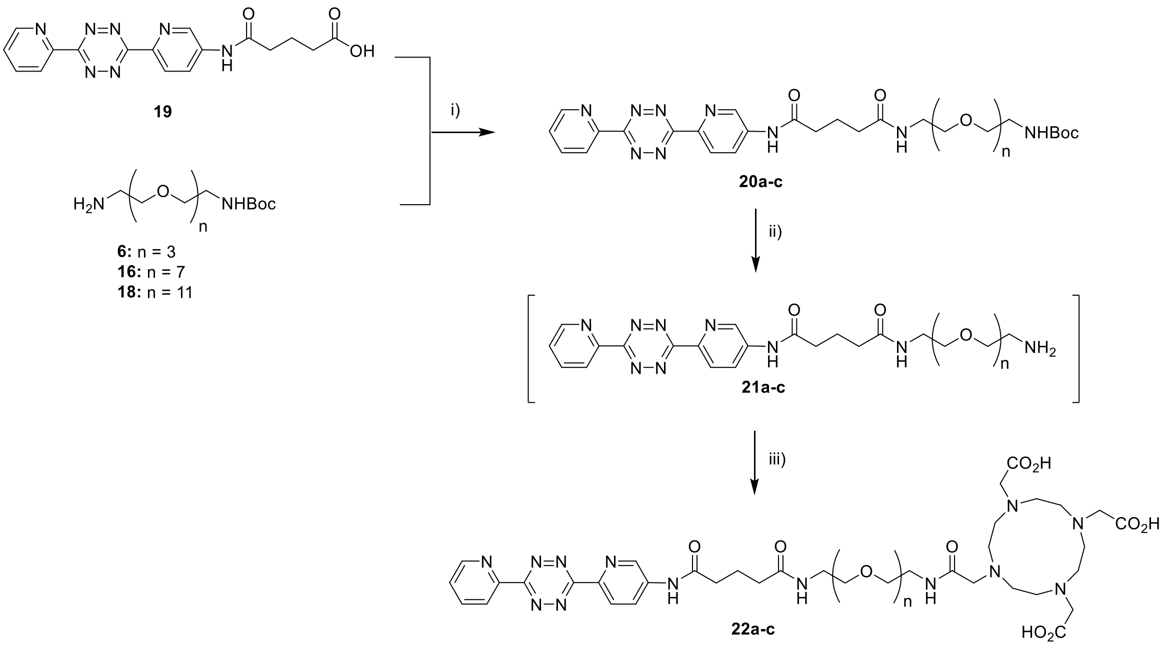


[a]**Reagents and conditions:** i) appropriate pegylated linker **6**, **16** or **18**, BOP, DMAP, DIPEA, DMF, rt, 12h; ii) TFA, $CH_2Cl_2$, -10°C then rt, 2h; iii) $CH_2Cl_2$, DOTA-NHS, DIPEA, rt, 16h.

Compound (**19**) was synthesized according to published procedure of Rossin *et al.*[13]

### 1.2.3.1. *tert*-Butyl (13,17-dioxo-17-((6-(6-(pyridin-2-yl)-1,2,4,5-tetrazin-3-yl)pyridin-3-yl)amino)-3,6,9-trioxa-12-azaheptadecyl)carbamate (20a)

To a solution of compound (**19**) (0.125 g, 0.34 mmol) in dry DMF at RT were successively added compound (**6**) (0.37 mmol), DIPEA (3.42 mmol) and benzotriazol-1-yloxy)tris(dimethylamino)phosphonium hexafluorophosphate (0.355 mmol). After stirring at RT for 16 h, the reaction mixture was evaporated under reduced pressure. The crude product was purified by column chromatography on silica gel using MeOH/$CH_2Cl_2$ (10/90, v/v) to afford compound (**20a**) (61%). $^1$H NMR (500 MHz, $CDCl_3$) $\delta$ 1.41 (s, 9H), 2.07-2.08 (m, 2H), 3.34-2.35 (m, 2H), 2.54-2.55 (m, 2H), 3.45-3.46 (m, 2H), 3.45-3.63 (m, 14H), 5.10 (brs, 1H), 6.46 (brs, 1H), 7.54-7.56 (m, 1H), 7.96-7.98 (t, 1H, *J* = 7.6 Hz), 8.56 (d, *J* = 8.4 Hz, 1H), 8.67-8.69 (m, 2H), 8.94-8.96 (m, 2H), 9.67 (brs, 1H); $^{13}$C NMR (125 MHz, $CDCl_3$) $\delta$ 21.5, 28.4, 35.0, 36.2, 39.3, 69.7, 70.0, 70.3, 70.4, 124.3, 125.2, 126.5, 126.8, 132.7, 137.5, 138.5, 142.0, 144.0, 150.2, 150.9, 163.3, 165.5, 171.4, HRMS for $C_{30}H_{41}N_9O_7$ m/z $[M+H]^+$ calc.: 640.3184; found: 640.3207.

[13]R. Rossin, P. R. Verkerk, S. M. van den Bosch, R. C. Vulders, I. Verel, J. Lub, M. S. Robillard. In vivo chemistry for pretargeted tumor imaging in live mice. *Angew. Chem. Int. Ed.*, **2010**, *49*, 19, 3375-3378.

**1.2.3.2. *tert*-Butyl (25,29-dioxo-29-((6-(6-(pyridin-2-yl)-1,2,4,5-tetrazin-3-yl)pyridin-3-yl)amino)-3,6,9,12,15,18,21-heptaoxa-24-azanonacosyl)carbamate (20b)**

To a solution of compound (**19**) (0.14 g, 0.38 mmol) in dry DMF at RT were successively added compound (**16**) (0.42 mmol), DIPEA (3.83 mmol) and benzotriazol-1-yloxy)tris(dimethylamino)phosphonium hexafluorophosphate (0.40 mmol). After stirring at RT for 16 h, the reaction mixture was evaporated under reduced pressure. The crude product was purified by column chromatography on silica gel using MeOH/$CH_2Cl_2$ (15/85, v/v) to afford compound (**20b**) (49%). $^1$H NMR (500 MHz, $CDCl_3$) $\delta$ 1.38 (s, 9H), 2.05 (quint, 2H, *J* = 6.9 Hz), 2.33 (t, 2H, *J* = 6.9 Hz), 2.55 (t, *J* = 6.9 Hz 2H), 3.41-3.42 (m, 2H), 3.46-3.48 (m, 2H), 3.52-3.58 (m, 28H), 5.08 (brs, 1H), 6.91 (brs, 1H), 7.51-7.54 (m, 1H), 7.96 (td, *J* = 7.8, 1.7 Hz, 1H), 8.57 (dd, *J* = 8.7, 2.4 Hz, 1H), 8.67 (m, 2H), 8.93 (m, 1H), 9.01 (d, *J* = 2.1 Hz, 1H), 10.04 (brs, 1H); $^{13}$C NMR (125 MHz, $CDCl_3$) $\delta$ 21.8, 28.5, 35.2, 36.4, 39.2, 40.6, 69.9, 70.0, 70.1, 70.3, 70.3, 70.4, 70.4, 70.6, 124.1, 125.3, 126.1, 127.1, 137.5, 138.9, 142.3, 143.6, 149.9, 151.0, 163.4, 163.5, 172.5, 173.4; HRMS for $C_{39}H_{51}N_9O_{11}$ m/z $[M+H]^+$ calc.: 830.4335; found: 830.4334.

**1.2.3.3. *tert*-Butyl (37,41-dioxo-41-((6-(6-(pyridin-2-yl)-1,2,4,5-tetrazin-3-yl)pyridin-3-yl)amino)-3,6,9,12,15,18,21,24,27,30,33-undecaoxa-36-azahentetracontyl)carbamate (20c)[13]**

To a solution of compound (**19**) (0.092 g, 0.25 mmol) in dry DMF were successively added compound (**18**) (0.28 mmol), DIPEA (2.51 mmol) and benzotriazol-1-yloxy)tris(dimethylamino)phosphonium hexafluorophosphate (0.26 mmol). After stirring at RT for 16 h, the reaction mixture was evaporated under reduced pressure. The crude product was purified by column chromatography on silica gel using MeOH/$CH_2Cl_2$ (15/85, v/v) to afford compound (**20c**) (67%). NMR $^1$H (500 MHz, $CDCl_3$) $\delta$ 1.37 (s, 9H), 2.02 (quint, *J* = 6.9 Hz, 2H), 2.31 (t, *J* = 6.9 Hz, 2H), 2.55 (t, *J* = 6.9 Hz, 2H), 3.36-3.43 (m, 2H), 3.46 (t, *J* = 5.1 Hz, 2H), 3.52-3.58 (m, 40H), 5.12 (brs, 1H), 6.91 (brs, 1H), 7.25-7.37 (m, 1H), 7.48-7.57 (m, 1H), 7.95 (td, *J* = 7.8, 1.7 Hz, 1H), 8.56 (dd, *J* = 8.7, 2.5 Hz, 1H), 8.67 (m, 2H), 8.89 (m, 1H), 8.97 (d, *J* = 2.4 Hz, 1H), 9.90 (brs, 1H). These data are in agreement with those of the literature.[13]

**1.2.3.4. 2,2',2''-(10-(2-Oxo-2-((2-(2-(5-oxo-5-((6-(6-(pyridin-2-yl)-1,2,4,5-tetrazin-3-yl)pyridin-3-yl)amino)pentanamido)ethoxy)ethyl)amino)ethyl)-1,4,7,10-tetraazacyclododecane-1,4,7-triyl)triacetic acid (22a)**

To a solution of compound (**20a**) (0.035g, 5.47 µmoles) in dry $CH_2Cl_2$, cooled to -10 °C, was added TFA (4.10 mmol). The resulting mixture was stirred at -10 °C for 2 h. The reaction mixture was evaporated under reduced pressure. The crude product was then co-evaporated twice with acetonitrile (5 mL) and diethyl ether (5 mL). The residue was dissolved in dry DMF solution and DIPEA (0.54 mmol) was added, followed by DOTA-NHS (0.136 mmol). The reaction mixture was stirred at RT for 14 h and then evaporated under reduced pressure. The crude product was purified by preparative HPLC on Combiflash® EZ Prep system (Teledyne Isco) on a RediSep® Prep C18 column (20 mm x 250 mm, 5 µm) eluted at a 15 mL/min flow rate with solvent A (Milli-Q water+0.1% TFA) and solvent B ($CH_3CN$+0.1% TFA) as followed: isocratic elution with 10% solvent B from 0 to 2 min, followed by two successive linear gradients from 10% to 50% solvent B over 16 min then from 50% to 100% solvent B over 1 min, and elution with 100% solvent B over 7 min ($\lambda$ 254 nm), thus providing after lyophilization compound (**22a**) ($R_T$ = 14.38 min, 75%). NMR $^1$H (500 MHz, DMSO-$d_6$) $\delta$ 1.23 (m, 2H), 2.13 (t, J = 7.4 Hz, 2H ), 2.41 (t, J = 7.4 Hz, 2H ), 3.09 (m, 6H), 3.21 (m, 3H), 3.24 (m, 3H), 3.37-3.42 (m, 9H), 3.47-3.49 (m, 9H), 7.77 (ddd, J = 7.6, 4.7, 1.1 Hz, 1H), 7.90 (t, J = 5.6 Hz), 8.14 (td, J = 7.8, 1.7 Hz), 8.39 (dd, J = 8.7, 2.5 Hz), 8.57 (d, J = 8.7 Hz, 1H), 8.60 (d, J = 8.7 Hz, 1H), 8.91 (m, 1H), 9.04 (d, J = 2.4 Hz, 1H), 10.53 (brs, 1H); HRMS for $C_{41}H_{59}N_{13}O_{12}$ m/z $[M+H]^+$ calc.: 463.7275; found: 463.7274. Afterwards, compound (**22a**) was

dissolved in Milli-Q water, so as to reach a 1 µg/µL concentration, and divided into aliquots containing 100 µg of Tz which were lyophilized. These aliquots were stored at -20 °C and were stable up to 6 months (control by analytical HPLC).

**1.2.3.5. 2,2',2''-(10-(2-Oxo-2-((2-(2-(5-oxo-5-((6-(6-(pyridin-2-yl)-1,2,4,5-tetrazin-3-yl)pyridin-3-yl)amino)pentanamido)ethoxy)ethyl)amino)ethyl)-1,4,7,10-tetraazacyclododecane-1,4,7-triyl)triacetic acid (22b)**

To a solution of compound (**20b**) (0.018g, 2.26 µmoles) in dry $CH_2Cl_2$, cooled to -10 °C, was added TFA (4.10 mmol). The resulting mixture was stirred at -10 °C for 2 h. The reaction mixture was evaporated under reduced pressure. The crude product was then co-evaporated twice with acetonitrile (5 mL) and diethyl ether (5 mL). The residue was dissolved in dry DMF solution and DIPEA (0.46 mmol) was added, followed by DOTA-NHS (5.51 µmoles). The reaction mixture was stirred at RT for 14 h and then evaporated under reduced pressure. The crude product was purified by preparative HPLC on Combiflash® EZ Prep system (Teledyne Isco) on a RediSep® Prep C18 column (20 mm x 250 mm, 5 µm) eluted at a 15 mL/min flow rate with solvent A (Milli-Q water+0.1% TFA) and solvent B ($CH_3CN$+0.1% TFA) as followed: isocratic elution with 10% solvent B from 0 to 2 min, followed by two successive linear gradients from 10% to 50% solvent B over 16 min then from 50% to 60% solvent B over 3 min ($\lambda$ 254 nm), thus providing after lyophilization compound (**22b**) ($R_T$ = 17.02 min, 58%). NMR $^1H$ (500 MHz, DMSO-$d_6$) $\delta$ 1.81 (m, 2H), 2.14 (t, J = 7.4 Hz, 2H ), 2.39 (t, J = 7.4 Hz, 2H ), 3.09 (m, 6H), 3.18 (m, 2H), 3.25 (m, 2H), 3.37-3.49 (m, 30H), 3.89 (m, 2H), 7.65 (m, 1H), 7.89 (m, 1H), 8.06 (td, J = 7.8, 1.7 Hz, 1H), 8.21 (d, J = 8.6 Hz, 1H), 8.32 (dd, J = 8.6, 2.4 Hz, 1H), 8.57 (d, J = 7.8 Hz, 1H), 8.79 (d, J = 4.7 Hz, 1H), 8.93 (d, J = 2.4 Hz, 1H), 10.53 (brs, 1H); HRMS for $C_{49}H_{75}N_{13}O_{16}$ m/z $[M+H]^+$ calc.: 551.2766; found: 551.2767. Afterwards, compound (**22b**) was dissolved in Milli-Q water, so as to reach a 1 µg/µL concentration, and divided into aliquots containing 100 µg of Tz which were lyophilized. These aliquots were stored at -20 °C and were stable up to 6 months (control by analytical HPLC).

**1.2.3.6. 2,2',2''-(10-(2-Oxo-2-((2-(2-(5-oxo-5-((6-(6-(pyridin-2-yl)-1,2,4,5-tetrazin-3-yl)pyridin-3-yl)amino)pentanamido)ethoxy)ethyl)amino)ethyl)-1,4,7,10-tetraazacyclododecane-1,4,7-triyl)triacetic acid (22c)**[13]

To a solution of compound (**20c**) (0.022 g, 2.22 µmoles) in dry $CH_2Cl_2$, cooled to -10°C, was added TFA (1.66 mmol). The resulting mixture was stirred at -10 °C for 2 h. The reaction mixture was evaporated under reduced pressure. The obtained crude product was then co-evaporated twice with acetonitrile (5 mL) and diethyl ether (5 mL). The residue was dissolved in dry DMF and DIPEA (0.46 mmol) was added, followed by DOTA-NHS (5.54 µmoles). The reaction mixture was stirred at RT for 14 h and then evaporated under reduced pressure. The crude product was purified by preparative HPLC on Combiflash® EZ Prep system (Teledyne Isco) on a RediSep® Prep C18 column (20 mm x 250 mm, 5 µm) eluted at a 15 mL/min flow rate with solvent A (Milli-Q water+0.1% TFA) and solvent B ($CH_3CN$+0.1% TFA) as followed: isocratic elution with 10% solvent B from 0 to 2 min, followed by two successive linear gradients from 10% to 50% solvent B over 16 min then from 50% to 60% solvent B over 3 min ($\lambda$ 254 nm), thus providing after lyophilization compound (**22c**) ($R_T$ = 21.05 min, 77%). NMR 1H (500 MHz, DMSO-d6) $\delta$ 1.81 (m, 2H), 2.14 (t, J = 7.4 Hz, 2H ), 2.39 (t, J = 7.4 Hz, 2H ), 3.09 (m, 6H), 3.18 (m, 2H), 3.25 (m, 2H), 3.37-3.49 (m, 30H), 3.89 (m, 2H), 7.65 (m, 1H), 7.89 (m, 1H), 8.06 (td, J = 7.8, 1.7 Hz, 1H), 8.21 (d, J = 8.6 Hz, 1H), 8.32 (dd, J = 8.6, 2.4 Hz, 1H), 8.57 (d, J = 7.8 Hz, 1H), 8.79 (d, J = 4.7 Hz, 1H), 8.93 (d, J = 2.4 Hz, 1H), 10.53 (brs, 1H); HRMS for $C_{57}H_{91}N_{13}O_{20}$ m/z $[M+H]^+$ calc.: 639.8324; found: 639.8336.

Afterwards, compound (**22c**) was dissolved in Milli-Q water, so as to reach a 1 µg/µL concentration, and divided into aliquots containing 100 µg of Tz which were lyophilized. These aliquots were stored at -20 °C and were stable up to 6 months (control by analytical HPLC).

## 2. Radiolabeling of TzPEG$_n$DOTA (Tz-1-4)

### 2.1. Materials for radiolabeling

[$^{177}$Lu]Lutetium solution was purchased from ITG (Germany) as a [$^{177}$Lu]$LuCl_3$ solution in 0.04 M aq. HCl. Water was distilled and deionized (18 MΩ/cm) by means of a Milli-Q water filtration system (Millipore). The labeling buffers were treated with Chelex-100 resin (BioRad Laboratories) overnight, then filtered through a "rapid flow" corning with a PES membrane (Thermofisher) and stored at 4 °C. ITLC-SG plates were purchased from Agilent Technologies. ITLC-SG were performed with citrate mobile phase (0.025 M, pH 5) and analyzed with Minigita DUAL radio-TLC scanner. Mobile phase (100 mL) was made up from a combination of citric acid (3.45 g) and sodium citrate (6.94 g) in MilliQ water. Each radiolabeling reaction was monitored by ITLC-SG (free $^{177}$Lu migrated to the solvent front while [$^{177}$Lu]Lu-Tz derivative remained at the baseline). The purifications by semi-preparative reversed phase-high pressure liquid chromatography (RP-HPLC) were performed on a Perkin Elmer system consisting of a Flexar LC autosampler, a series 200 pump, a Peltier column oven, a vacuum degasser and a PDA and GabiStar Raytest detectors. Semi-preparative RP-HPLC purifications were carried out on a Waters SymmetryPrep™ C18 column (7.8 mm × 300 mm, 7 µm) using the following conditions: flow rate: 2 mL/min, eluent A (Milli-Q water+0.1% TFA), eluent B ($CH_3CN$+0.1% TFA) and detection wavelengths set at 254 and 330 nm. (1) For TzPEG$_4$ derivative, the elution starts with 10% eluent B (0 to 0.5 min), followed by two linear gradients, firstly 10% to 22% eluent B from 0.5 to 20 min, and secondly from 22% to 100% eluent B over 5 min, an isocratic elution with 100% B for 4 min, and finally returned to 10% B over 0.5 min. (2) For TzPEG$_8$ derivative, the elution begins with 20% eluent B from 0 to 2 min, followed by two successive linear gradients, firstly from 20% to 50% B over 23 min, and secondly from 50% to 100% B over 5 min; 100 % B is kept for 30 to 35 min, and finally returned to 20 % B for 35 to 40 min. Analytical RP-HPLC analyses were performed on an Agilent series 1100 HPLC system equipped with an online degasser, a quaternary pump, an automatic sampler, a DAD detector and coupled to a 500TR series (ULTIMO-FLO™). Analytical RP-HPLC analyses were carried out on an Agilent Zorbax extend C18 column (4.6 mm × 150 mm, 5 µm) using the following conditions: analysis time: 20 min, flow rate: 1 mL/min, eluent A (Milli-Q water+0.1% TFA), eluent B ($CH_3CN$+0.1% TFA) and detection wavelengths set at 254 nm and 330 nm. The elution program for TzPEG$_4$ derivative starts with an isocratic elution with 25% eluent B for 3.5 min, followed by a linear gradient from 25% to 50% eluent B over 3.5 min and an isocratic elution with 50% eluent B for 13 min. The optimized elution program for TzPEG$_8$ or TzPEG$_{12}$ derivatives consists of an isocratic elution with 5% eluent B for 3.5 min, followed by a linear gradient from 5% to 50% eluent B for 3.5 min, and ends with an isocratic elution with 50% B for 13 min. The molar activity ($A_m$) was calculated as following [(%radiochemical purities)/(numbers of moles of cold compound)]*radioactivity, where numbers of moles and radioactivity were expressed in µmol and GBq, respectively. For method A, number of moles of "cold compound" was determined using calibration curve by integrating the corresponding peak detected by UV obtained by analytical chromatography RP-HPLC. The radioactivity corresponds to the radioactivity injected into HPLC. For Method B, number of moles of "cold compound" refers to the initial quantity of precursor and radioactivity corresponds to the final radioactivity after formulation.

**Radiolabeling of $TzPEG_nDOTA$ derivatives**

*Method A :* To a solution of $TzPEG_nDOTA$ derivative (Tz-**1-3**) (100 µg) in NaOAc buffer (0.025 M, pH 7.0, 100 µL) was added a suitable amount of $[^{177}Lu]LuCl_3$ in 0.04 M HCl (40-500 MBq) and the reaction mixture was heated at 50 °C until completion of the reaction (20 min, ITLC monitoring). The reaction mixture was purified by semi-preparative (radio)-RP-HPLC as mentioned above. The collected radiotracer fraction was evaporated under reduced pressure. The residue was diluted with Milli-Q water (20 mL) and purified by adsorption on a Sep-Pak C18 light cartridge. Elution with 0.6 mL of EtOH followed by concentration under reduced pressure afforded the desired radiotracer which was then formulated in sterile isotonic saline. The final solution contained less than 10 % of EtOH (v/v). The identity and radiochemical purity of radiotracer was confirmed by analytical radio-RP-HPLC. Molar activity (MA) was determined on the basis of a UV/mass calibration curve carried out under analytical radio-RP-HPLC conditions using chromatograms recorded at 330 nm.

*Method B :* To a solution of $TzPEG_nDOTA$ derivative (Tz-**1-4**) (25µL of 100 µg/100 µL aliquot in DMSO) in NaOAc buffer (0.25 M, pH 5.5, 200 µL) was added a suitable amount of $[^{177}Lu]LuCl_3$ in 0.04 M HCl (40-500 MBq) and the reaction mixture was kept at RT until completion of the reaction (10 min, ITLC). The reaction mixture was then diluted with Milli-Q water (20 mL) and purified by adsorption on a Sep-Pak C18 light cartridge (Waters, Milford, MA, USA). Elution with 0.6 mL of ethanol followed by concentration under reduced pressure provided the desired radiotracer which was then formulated in sterile isotonic saline. The final solution contained less than 10 % of EtOH (v/v). The identity and radiochemical purity of radiotracer were confirmed by analytical radio-RP-HPLC. Molar activity (MA) was determined on the basis of a UV/mass calibration curve carried out under analytical radio-RP-HPLC conditions using chromatograms recorded at 330 nm.

### 2.1.1. $TzPEG_4DOTA$-$[^{177}Lu]$ ($[^{177}Lu]$Lu-Tz-1)

*Method A:* retention time for semi-preparative purification ($R_t$ = 14.38 min); retention time for analytical control ($R_t$ = 10.1 min); RCY = 60-80% (uncorrected); radiochemical purity: 98%; $A_m$ > 8 GBq/µmol.

*Method B:* retention time for analytical control ($R_t$ = 10.1 min); RCY = 79%; radiochemical purity > 99%; MA = 12.2 GBq/µmol.

### 2.1.2. $TzPEG_8DOTA$-$[^{177}Lu]$ ($[^{177}Lu]$Lu-Tz-2)

*Method A:* retention time for semi-preparative purification ($R_t$ = 17.02 min); retention time for analytical control ($R_t$ = 13.1 min); RCY = 36%, radiochemical purity: 28%; $A_m$ = nd

*Method B:* retention time for analytical control ($R_t$ = 13.1 min); RCY = 89%; radiochemical purity: 99%; $A_m$ = 9.3 GBq/µmol

### 2.1.3. TzPEG$_{12}$DOTA-[$^{177}$Lu] ([$^{177}$Lu]Lu-Tz-3)

Method B: retention time for analytical control ($R_t$ = 13.5 min); RCY = 88%; radiochemical purity: 97%; $A_m$ = 7.4 GBq/µmol.

### 2.1.4. TzPEG$_8$DOTA-[$^{177}$Lu] ([$^{177}$Lu]Lu-Tz-4)

Method B: retention time for analytical control ($R_t$ = 13.5 min); RCY = 93%; radiochemical purity: >99%; $A_m$ = 13.9 GBq/µmol.

## 3. Biodistribution experiments

*Influence of the injection routes of 35A7-TCO*: 30 mice were assigned to three groups (n= 9 or 12 *per* group). 50 µg of 35A7-TCO (≈ 3-4 TCO *per* mAb) were either i.v. (*i.e*. group 1, n=9) or i.p. (*i.e*. group 2, n=9) injected followed 24 h later by i.p. injection of 10 MBq of [$^{177}$Lu]Lu-Tz-**1**. In these groups, mice were sacrificed at 24 h, 48 h and 144 h after injection. Control group (*i.e*. group 3, n=12) received a single i.p. injection of [$^{177}$Lu]Lu-Tz-**1**. In this group, mice were sacrificed at 5 min, 1 h, 3 h and 24 h after injection. After all mouse sacrifices, peritoneal carcinomatosis tumors and main organs (*i.e*. blood, heart, liver, kidneys, caecum, colon, muscle and bone marrow) were harvested and counted in a gamma counter (1480 automatic gamma counter, Wallac Wizard 3", PerkinElmer, France).

- **4. Determination of [$^{177}$Lu]Lu-Tz-1 hematologic toxicity**

*Hematocrit and hemoglobin.* An average of 70 µL of blood was collected from mouse tail vein, 3 and 10 days after injections, using heparinized capillaries. After 15 min of centrifugation (1000 g, TA), hematocrit (%) is determined as the ratio of the volume of red blood cells to the total volume of blood. Hemoglobin (g/L) is calculated using the formula [hemoglobin = hematocrit /3].

*Leucocytes counting*. A precise amount of 20 µL of blood was collected from mouse tail vein, 3 and 10 days after injections, using non-heparinized capillaries. Blood was then lysed in tubes containing blue reagent (*in vitro* kit Leuko-TIC, Bioanalytic GmbH), coloring leukocytes in blue thus allowing their counting using a phase contrast microscope.

**Supplementary Figure S1**: Influence of the injection route of 35A7-TCO on the interaction towards [$^{177}$Lu]LuTz-**1** in mice bearing A431-CEA-Luc disseminated tumors. (A) Left: SPECT-CT imaging of [$^{177}$Lu]LuTz-**1** (10 MBq) at 3 h and 24 h p.i., without prior injection of 35A7-TCO. Right: %IA/g in organs at different time p.i. (B) Left: SPECT-CT imaging of [$^{177}$Lu]LuTz-**1** (10 MBq) 3 h and 24 h post injection, with prior injection of 50 µg of 35A7-TCO either i.v. (top) or i.p. (bottom) 24 h before. Right: Comparison of the %IA/g measured in organs at different time points according to 35A7-TCO injection route. B: bladder, I: intestines, T: tumors. Scale bars SPECT: Max: 2500 kBq/mL, Min: 700 kBq/mL (3 h) and Max: 4000 kBq/mL, Min: 0 kBq/mL (24 h); CT: Max: 4000 HU, Min: 800 HU (at both 3 h and 24 h).

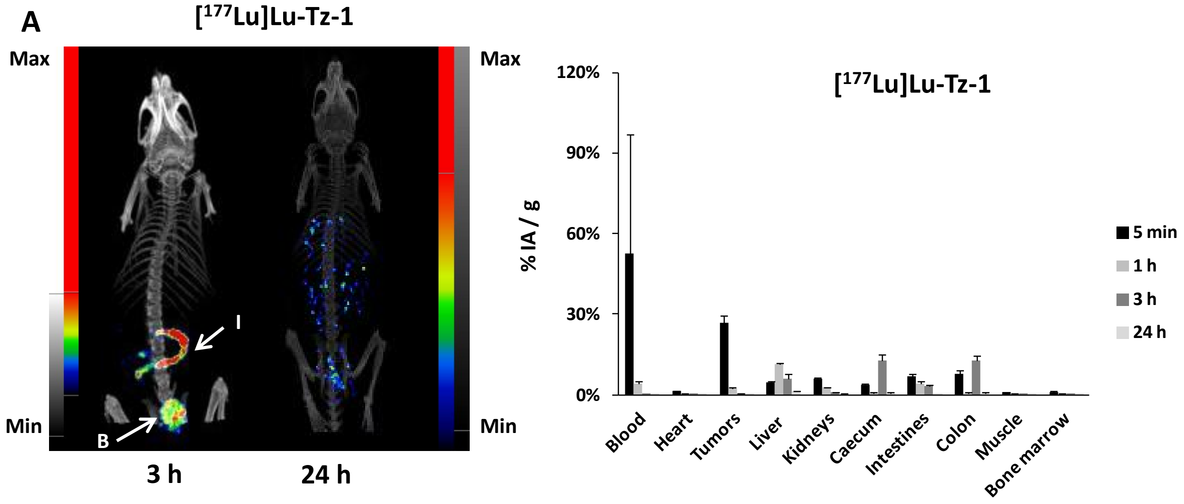

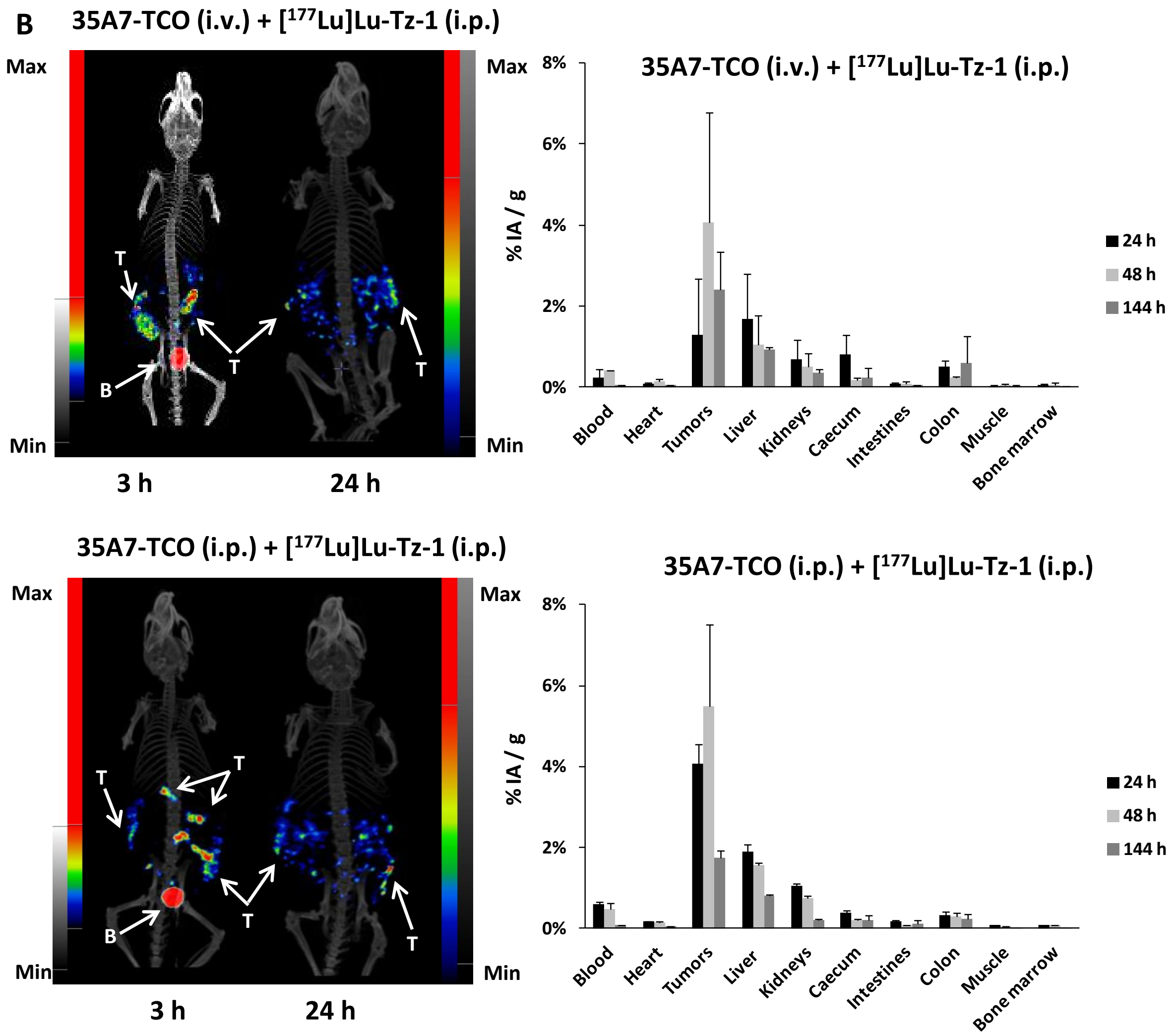
B
35A7-TCO (i.v.) + [177Lu]Lu-Tz-1 (i.p.)
Max
Min
T
B
T
T
3 h
24 h
35A7-TCO (i.v.) + [177Lu]Lu-Tz-1 (i.p.)
% IA / g
8%
6%
4%
2%
0%
Blood
Heart
Tumors
Liver
Kidneys
Caecum
Intestines
Colon
Muscle
Bone marrow
24 h
48 h
144 h
35A7-TCO (i.p.) + [177Lu]Lu-Tz-1 (i.p.)
Max
Min
T
T
B
T
T
3 h
24 h
35A7-TCO (i.p.) + [177Lu]Lu-Tz-1 (i.p.)
% IA / g
8%
6%
4%
2%
0%
Blood
Heart
Tumors
Liver
Kidneys
Caecum
Intestines
Colon
Muscle
Bone marrow
24 h
48 h
144 h

**Supplementary Figure S2**: Control of the tumor uptake in mice and the tumor growth medians of the different groups after mice randomization using bioluminescence imaging.

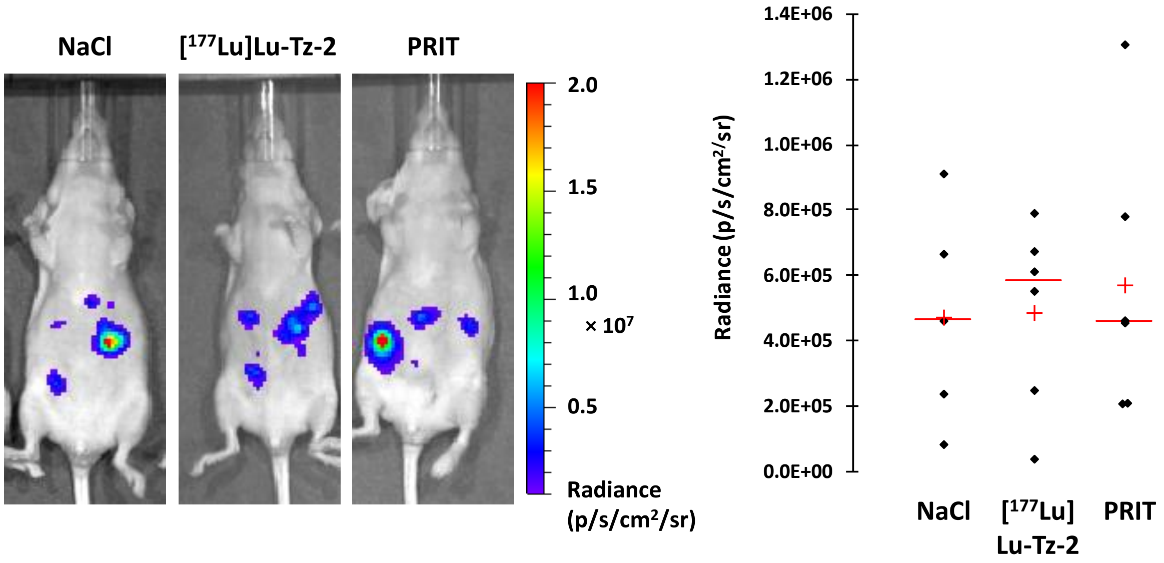

**Supplementary Figure S3:** Determination of the hematologic toxicity induced by intraperitoneal injections of [177Lu]Lu-Tz-1 alone or with prior injection of 35A7-TCO (PRIT). (A) Hematocrit measurement. Basal hematocrit in healthy mice ranges from 50 to 60%.[14] (B) Calculation of the corresponding hemoglobin. (C) Number of leucocytes counted. N = 6 mice per group. P value is considered significant when < 0.05 (Two-way ANOVA).

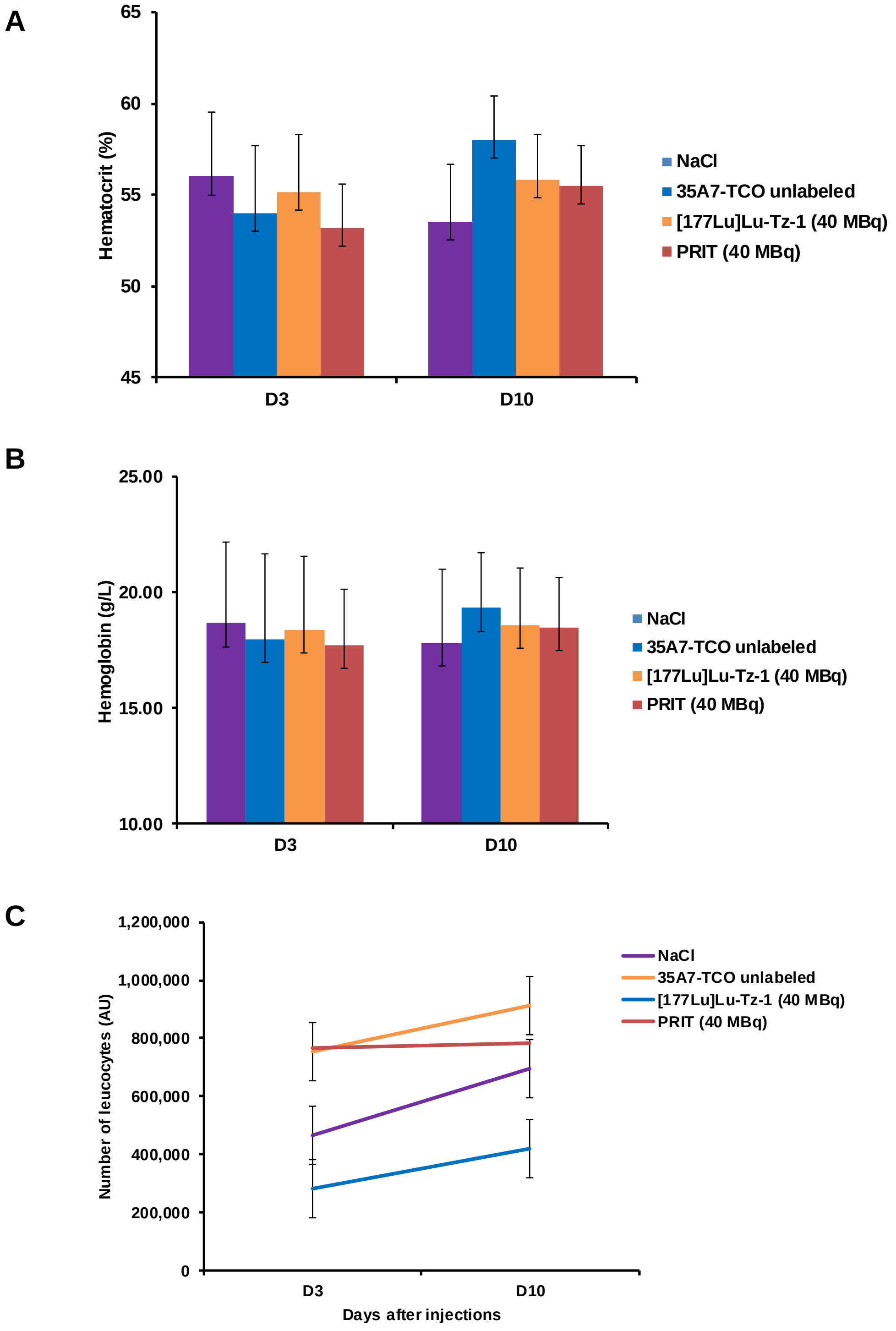


[14] Provencher-Bolliger A., Everds N., The laboratory mouse, **2012**, 2nd Edition.

**Supplementary Figure S4**: Radio-HPLC chromatograms of formulated radiolabeled [$^{177}$Lu]Lu-Tz-**1-4**

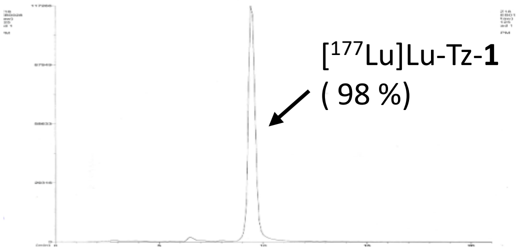


Method A

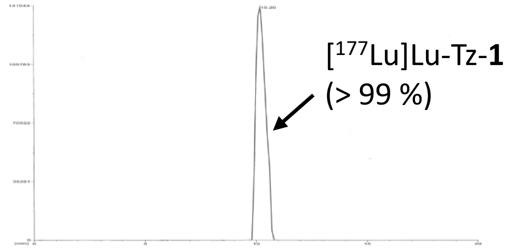


Method B

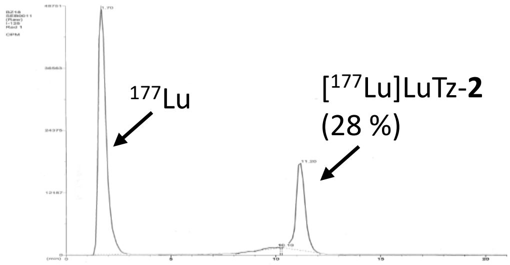


Method A

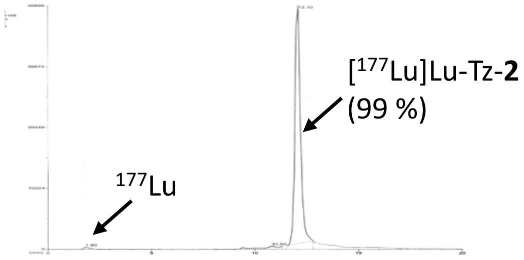


Method B

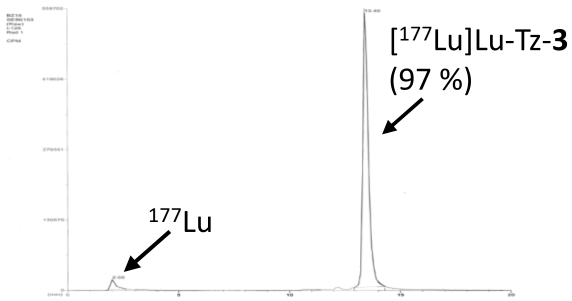


Method B

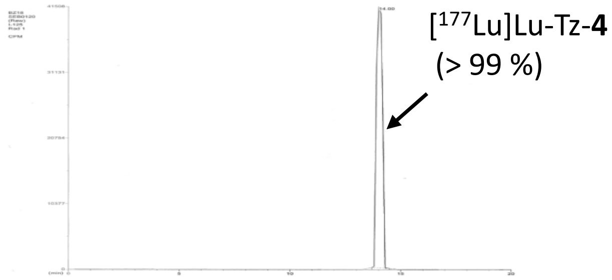


Method B

**Supplementary Table S1**: %IA/g of [$^{177}$Lu]Lu-Tz-**1-3** (10 MBq) 24 h post injection. Values are mean ± SEM.

| Organ | [$^{177}$Lu]Lu-Tz-1 | [$^{177}$Lu]Lu-Tz-2 | [$^{177}$Lu]Lu-Tz-3 |
|---|---|---|---|
| **Blood** | 0.02 ± 0.001 | 0.22 ± 0.022 | 0.03 ± 0.040 |
| **Heart** | 0.02 ± 0.001 | 0.45 ± 0.010 | 0.02 ± 0.015 |
| **Tumors** | 0.33 ± 0.138 | 0.28 ± 0.193 | 0.30 ± 0.120 |
| **Liver** | 1.47 ± 0.066 | 0.20 ± 0.010 | 0.11 ± 0.231 |
| **Kidneys** | 0.70 ± 0.052 | 0.50 ± 0.053 | 0.57 ± 0.215 |
| **Intestines** | 0.07 ± 0.003 | 0.10 ± 0.037 | 0.05 ± 0.026 |
| **Caecum** | 0.23 ± 0.045 | 0.19 ± 0.084 | 0.14 ± 0.019 |
| **Colon** | 0.19 ± 0.023 | 0.29 ± 0.032 | 0.10 ± 0.068 |
| **Muscle** | 0.01 ± 0.003 | 0.11 ± 0.004 | 0.01 ± 0.005 |
| **Bone marrow** | 0.09 ± 0.007 | 0.23 ± 0.027 | 0.20 ± 0.009 |

**Supplementary Table S2**: %IA/g of [$^{177}$Lu]Lu-Tz-**1-3** (10 MBq) measured on the entire abdomen 24 h post injection using SPECT imaging. Values are mean ± SEM.

| Probe | Abdomen |
|---|---|
| **[$^{177}$Lu]Lu-Tz-1** | 15.66 ± 1.880 |
| **[$^{177}$Lu]Lu-Tz-2** | 4.72 ± 2.010 |
| **[$^{177}$Lu]Lu-Tz-3** | 2.05 ± 0.176 |

**Supplementary Table S3**: %IA/g of [$^{177}$Lu]Lu-Tz-**1-4** (10 MBq) (with prior injection of 50 µg of 35A7-TCO 24 h before) 24 h, 48 h and 144 h post injection. Values are mean ± SEM.

| | Organ | [$^{177}$Lu]Lu-Tz-1 | [$^{177}$Lu]Lu-Tz-2 | [$^{177}$Lu]Lu-Tz-3 | [$^{177}$Lu]Lu-Tz-4 |
|---|---|---|---|---|---|
| **24 h post injection** | **Blood** | 0.46 ± 0.091 | 0.28 ± 0.045 | 0.15 ± 0.076 | 0.36 ± 0.046 |
| | **Heart** | 0.15 ± 0.014 | 0.12 ± 0.007 | 0.07 ± 0.023 | 0.15 ± 0.011 |
| | **Tumors** | 4.00 ± 2.667 | 6.29 ± 4.270 | 3.48 ± 1.199 | 8.88 ± 5.606 |
| | **Lungs** | 0.22 ± 0.014 | 0.17 ± 0.016 | 0.25 ± 0.264 | 0.21 ± 0.016 |
| | **Liver** | 1.53 ± 0.252 | 0.27 ± 0.122 | 0.22 ± 0.087 | 0.28 ± 0.012 |
| | **Kidneys** | 0.67 ± 0.043 | 0.76 ± 0.136 | 0.65 ± 0.284 | 0.65 ± 0.010 |
| | **Intestines** | 0.12 ± 0.022 | 0.12 ± 0.028 | 0.09 ± 0.034 | 0.09 ± 0.005 |
| | **Caecum** | 0.31 ± 0.087 | 0.34 ± 0.296 | 0.14 ± 0.091 | 0.24 ± 0.036 |
| | **Colon** | 0.26 ± 0.053 | 0.36 ± 0.340 | 0.11 ± 0.049 | 0.27 ± 0.053 |
| | **Muscle** | 0.05 ± 0.007 | 0.08 ± 0.065 | 0.03 ± 0.013 | 0.04 ± 0.001 |
| | **Bone marrow** | 0.09 ± 0.007 | 0.20 ± 0.132 | 0.33 ± 0.293 | 0.10 ± 0.006 |

| | Organ | [$^{177}$Lu]Lu-Tz-1 | [$^{177}$Lu]Lu-Tz-2 | [$^{177}$Lu]Lu-Tz-3 | [$^{177}$Lu]Lu-Tz-4 |
|---|---|---|---|---|---|
| **48 h post injection** | **Blood** | 0.22 ± 0.063 | 0.42 ± 0.224 | 0.23 ± 0.132 | 0.36 ± 0.151 |
| | **Heart** | 0.07 ± 0.035 | 0.11 ± 0.032 | 0.08 ± 0.032 | 0.13 ± 0.016 |
| | **Tumors** | 3.47 ± 1.626 | 5.08 ± 4.412 | 4.59 ± 2.134 | 7.46 ± 5.255 |
| | **Lungs** | 0.16 ± 0.035 | 0.17 ± 0.049 | 0.17 ± 0.114 | 0.21 ± 0.037 |
| | **Liver** | 1.10 ± 0.024 | 0.29 ± 0.048 | 0.19 ± 0.036 | 0.26 ± 0.012 |
| | **Kidneys** | 0.52 ± 0.027 | 0.57 ± 0.039 | 0.52 ± 0.136 | 0.43 ± 0.012 |
| | **Intestines** | 0.12 ± 0.040 | 0.08 ± 0.017 | 0.05 ± 0.007 | 0.11 ± 0.037 |
| | **Caecum** | 0.48 ± 0.377 | 0.11 ± 0.054 | 0.09 ± 0.031 | 0.11 ± 0.019 |
| | **Colon** | 0.49 ± 0.385 | 0.12 ± 0.035 | 0.10 ± 0.035 | 0.13 ± 0.021 |
| | **Muscle** | 0.03 ± 0.003 | 0.05 ± 0.008 | 0.04 ± 0.020 | 0.10 ± 0.069 |
| | **Bone marrow** | 0.06 ± 0.008 | 0.22 ± 0.119 | 0.27 ± 0.226 | 0.08 ± 0.008 |

| 144 h post injection | Organ | [$^{177}$Lu]Lu-Tz-1 | [$^{177}$Lu]Lu-Tz-2 | [$^{177}$Lu]Lu-Tz-3 | [$^{177}$Lu]Lu-Tz-4 |
|---|---|---|---|---|---|
| | **Blood** | 0.04 ± 0.012 | 0.07 ± 0.033 | 0.06 ± 0.055 | 0.04 ± 0.035 |
| | **Heart** | 0.03 ± 0.004 | 0.04 ± 0.011 | 0.03 ± 0.013 | 0.02 ± 0.016 |
| | **Tumors** | 1.50 ± 0.605 | 3.80 ± 2.183 | 1.52 ± 0.932 | 5.56 ± 3.496 |
| | **Lungs** | 0.04 ± 0.003 | 0.06 ± 0.008 | 0.05 ± 0.019 | 0.04 ± 0.029 |
| | **Liver** | 0.57 ± 0.118 | 0.15 ± 0.038 | 0.13 ± 0.026 | 0.11 ± 0.070 |
| | **Kidneys** | 0.17 ± 0.022 | 0.17 ± 0.009 | 0.16 ± 0.020 | 0.11 ± 0.052 |
| | **Intestines** | 0.02 ± 0.005 | 0.03 ± 0.015 | 0.02 ± 0.009 | 0.02 ± 0.010 |
| | **Caecum** | 0.03 ± 0.002 | 0.05 ± 0.024 | 0.03 ± 0.023 | 0.03 ± 0.011 |
| | **Colon** | 0.03 ± 0.001 | 0.05 ± 0.021 | 0.04 ± 0.028 | 0.03 ± 0.019 |
| | **Muscle** | 0.03 ± 0.010 | 0.01 ± 0.002 | 0.01 ± 0.003 | 0.01 ± 0.005 |
| | **Bone marrow** | 0.02 ± 0.011 | 0.23 ± 0.142 | 0.33 ± 0.225 | 0.03 ± 0.018 |
